# The steroid hormone 20-hydroxyecdysone counteracts insulin signaling *via* insulin receptor dephosphorylation

**DOI:** 10.1016/j.jbc.2021.100318

**Published:** 2021-01-20

**Authors:** Yan-Li Li, You-Xiang Yao, Yu-Meng Zhao, Yu-Qin Di, Xiao-Fan Zhao

**Affiliations:** Shandong Provincial Key Laboratory of Animal Cells and Developmental Biology, School of Life Sciences, Shandong University, Qingdao, China

**Keywords:** insulin, insulin receptor, 20-hydroxyecdysone, PTP1B, PTEN, FoxO, hemolymph glucose, body growth, metamorphosis, 20E, 20-hydroxyecdysone, λPPase, lambda protein phosphatase, ACTB/β-actin, actin beta, AKT, protein kinase B, Co-IP, co-immunoprecipitation, ChIP, chromatin immunoprecipitation, DAPI, 4’-6-diamidino-2-phenylindole dihydrochloride, DILP6, *Drosophila melanogaster* insulin-like peptide 6, DMSO, dimethyl sulfoxide, DopEcR, dopamine receptor, DPBS, dulbecco’s phosphate-buffered saline, dsRNA, double-stranded RNA, E20MO, Ecdysone 20-monooxygenase, FBS, fetal bovine serum, FoxO, Forkhead box O, FoxOBE, FoxO-binding element, GFP, green fluorescent protein, GLUT4, glucose transporter 4, HaEpi, *Helicoverpa armigera* epidermal cell line, IIS, insulin/insulin-like growth factor-1 (IGF-1) signaling, IGFs, insulin-like growth factors, IgG, immunoglobin G, ILPs, insulin-like peptides, INSR, insulin receptor, INSRβ, non-Phospho-INSRβ, ORF, open reading frame, p-INSRβ, Phospho-INSRβ, PBS, phosphate-buffered saline, PCD, programmed cell death, PDK1, phosphoinositide-dependent protein kinase 1, PG, prothoracic gland, PI3K, phosphoinositide-3-kinase, PIP2, phosphatidylinositol 4,5-diphosphate, PIP3, phosphatidylinositol 3,4,5-triphosphate, PTEN, PTEN/MMAC1/TEP1 (phosphatase and tensin homolog deleted on chromosome 10/mutated in multiple advanced cancer 1/TGF-regulated and epithelial cell-enriched phosphatase), PTP1B, protein tyrosine phosphatase 1B, PTPase, tyrosine–protein phosphatase, qRT-PCR, quantitative real-time reverse transcription PCR, RNAi, RNA interference, SDS-PAGE, sodium dodecyl sulfate–polyacrylamide gel electrophoresis, T2D, Type 2 diabetes

## Abstract

The insulin receptor (INSR) binds insulin to promote body growth and maintain normal blood glucose levels. While it is known that steroid hormones such as estrogen and 20-hydroxyecdysone counteract insulin function, the molecular mechanisms responsible for this attenuation remain unclear. In the present study, using the agricultural pest lepidopteran *Helicoverpa armigera* as a model, we proposed that the steroid hormone 20-hydroxyecdysone (20E) induces dephosphorylation of INSR to counteract insulin function. We observed high expression and phosphorylation of INSR during larval feeding stages that decreased during metamorphosis. Insulin upregulated INSR expression and phosphorylation, whereas 20E repressed INSR expression and induced INSR dephosphorylation *in vivo*. Protein tyrosine phosphatase 1B (PTP1B, encoded by *Ptpn1*) dephosphorylated INSR *in vivo*. PTEN (phosphatase and tensin homolog deleted on chromosome 10) was critical for 20E-induced INSR dephosphorylation by maintaining the transcription factor Forkhead box O (FoxO) in the nucleus, where FoxO promoted *Ptpn1* expression and repressed *Insr* expression. Knockdown of *Ptpn1* using RNA interference maintained INSR phosphorylation, increased 20E production, and accelerated pupation. RNA interference of *Insr* in larvae repressed larval growth, decreased 20E production, delayed pupation, and accumulated hemolymph glucose levels. Taken together, these results suggest that a high 20E titer counteracts the insulin pathway by dephosphorylating INSR to stop larval growth and accumulate glucose in the hemolymph.

Insulin, insulin-like growth factors (IGFs), and insulin-like peptides (ILPs) promote growth *via* insulin/IGF signaling (IIS) (1). The steroid hormones 20-hydroxecdysone (20E) and estrogen attenuate insulin signaling and the growth rate in *Drosophila* and humans, respectively (2). Insulin and 20E are the main regulators of insect growth (3). The insulin pathway determines the growth rate, and 20E determines the duration of growth (4). However, despite intensive research, how animals regulate growth and growth termination by the cross talk between insulin and steroid hormones remains unclear.

In addition, alterations of the insulin pathway also result in diabetes *via* insulin insufficiency (type I diabetes) or insulin resistance and pancreatic β-cell dysfunction (type II diabetes) (5, 6). Insulin maintains normal blood glucose levels; however, the steroid hormones counteract insulin function and increase blood glucose levels, even cause diabetes (7). For example, glucocorticoids, which are widely used anti-inflammatory and immunosuppressive drugs (5), induce hyperglycemia and insulin-resistant diabetes (8); however, the mechanisms are not fully understood. The regulation of hemolymph glucose levels by 20E and its mechanism are also unclear.

The insulin receptor (INSR) is a receptor tyrosine kinase that plays important roles in the insulin pathway by binding its ligand (insulin) to regulate glucose, fatty acids, and protein metabolism to promote growth (9). INSR is a constitutive homodimeric transmembrane glycoprotein (10), comprising two α and two β subunits linked by disulfide bridges (11). INSR is encoded by the *Insr* gene as a single protein. A protease, furin, cleaves the protein into the α and β subunits, named INSRα and INSRβ, respectively (12). INSRα has insulin-binding sites and is located outside the cell membrane. INSRβ contains a transmembrane domain and the intracellular tyrosine kinase elements (13). Insulin binding causes a conformational change and autophosphorylation of INSRβ, resulting in phosphorylation of phosphoinositide-3-kinase (PI3K), which phosphorylates phosphatidylinositol 4, 5-diphosphate (PIP2) to phosphatidylinositol 3,4,5-triphosphate (PIP3) (14). PIP3 attracts AKT/protein kinase B (PKB) to the cell membrane, where it is phosphorylated by the phosphoinositide-dependent protein kinase 1 (PDK1) (9). AKT phosphorylates AS160 protein, which promotes glucose transporter 4 (GLUT4) translocation to the cell membrane for glucose uptake into the cell from blood (9). AKT also phosphorylates Forkhead box O (FoxO), a negative regulator of the insulin pathway, to locate FoxO in the cytoplasm, thus blocking its transcriptional activity in the nucleus (15). The above insulin-induced events can be reversed by the pathway's negative regulator, phosphatase, and tensin homolog deleted on chromosome 10 (PTEN), also named as MMAC1 (mutated in multiple advanced cancer 1), or TEP1 (TGF-regulated and epithelial cell-enriched phosphatase) (16). The INSR-mediated insulin signaling pathway and its downstream signaling molecules are structurally and functionally conserved throughout evolution from worms to mammals (17).

Insulin promotes growth and ecdysone production in the prothoracic gland (PG), and in turn, the increased 20E level counteracts insulin function and promotes insect metamorphosis (18). 20E regulates the nuclear localization of transcription factor FoxO to counteract the role of insulin in promoting growth in *Drosophila melanogaster* (19). In *Helicoverpa armigera*, 20E upregulates *Pten* expression to repress AKT phosphorylation; therefore, FoxO cannot be phosphorylated by AKT and enters the nucleus to induce downstream gene transcription in the 20E pathway (20). In addition, a high 20E titer inhibits *Pdk1* expression and represses AKT and FoxO phosphorylation, resulting in FoxO nuclear localization to induce autophagy and repress cell proliferation in *H. armigera* (21). All these studies indicate that 20E counteracts the insulin pathway; however, whether 20E exerts this effect from the beginning of the insulin pathway by repressing INSR phosphorylation and expression, and the consequence, is unknown.

To understand the mechanisms by which steroid hormones counteract the insulin pathway, we used the lepidopteran insect *H. armigera*, an agricultural pest, as a model, and the well-known factors in insulin pathway as readouts for the study. Our study revealed that INSR plays roles in insect larval growth and therefore promotes 20E production to reach a critical titer during larval feeding stages. 20E induces the dephosphorylation of INSR to repress the insulin pathway. Thus, the steroid hormone 20E counteracts the insulin pathway to stop larval growth and accumulate hemolymph glucose.

## Results

### The expression and phosphorylation levels of INSRβ were decreased during metamorphosis

**Figure 1:**
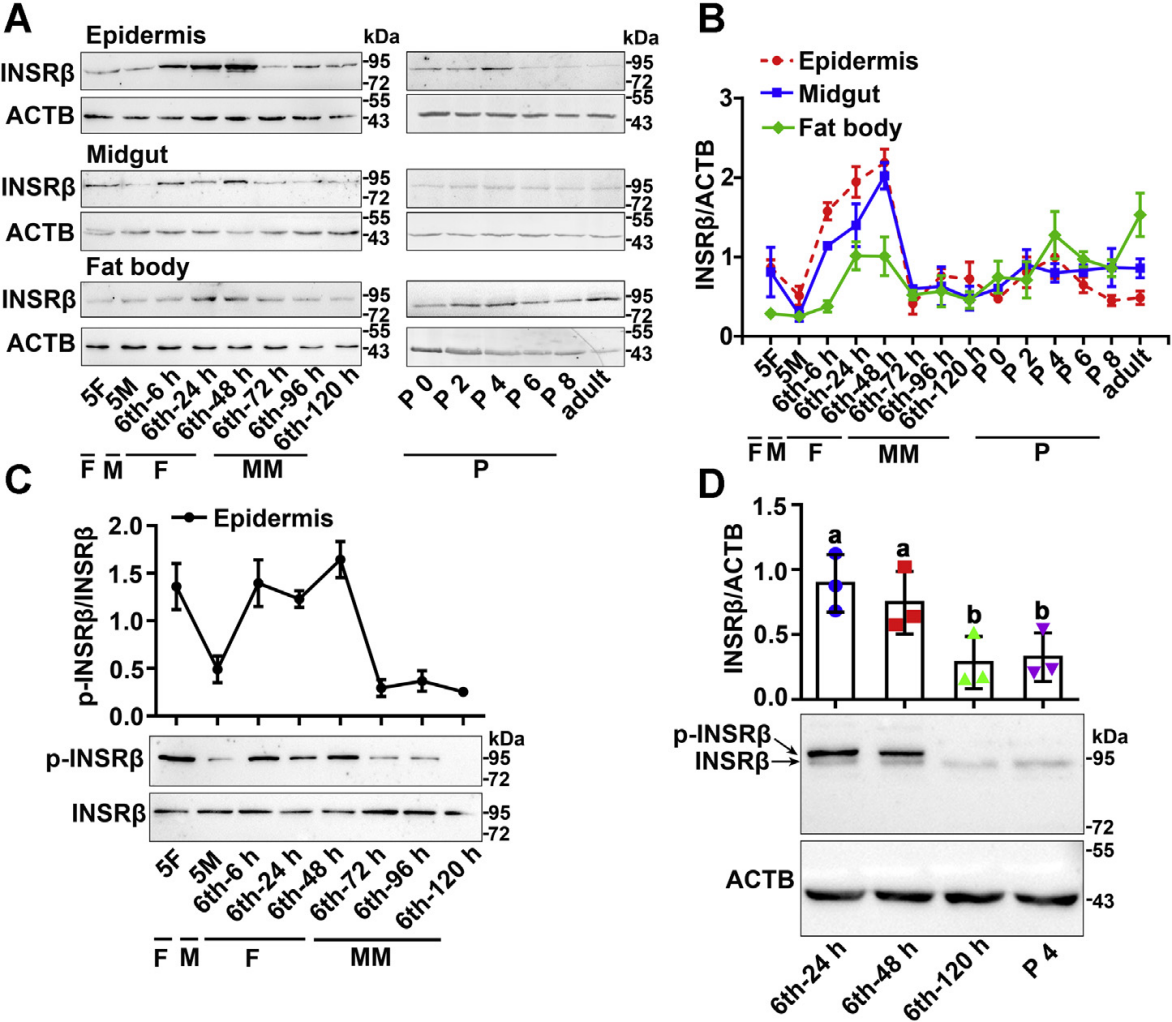
**Western blotting showing the decrease in INSRβ levels and phosphorylation during metamorphosis**. *A*, the expression levels of INSRβ in tissues detected by antibody against INSRβ. 5F, fifth instar feeding larvae; 5M, fifth instar molting larvae; sixth-6 h–sixth-120 h, time stages of sixth instar larvae; P 0–P 8, 0-day-old to 8-day-old pupae; Adult, adult; F, feeding; M, molting; MM, metamorphic molting; P, pupae stage. ACTB was detected by the monoclonal antibody ACTB Rabbit mAb as the internal control. The loading quantity for each lane is 50 μg proteins. 12.5% SDS-PAGE gel. *B*, quantitative analysis of the INSRβ expression using ImageJ software. *C*, variation of INSRβ phosphorylation levels in epidermis during larval development detected by antibody against p-INSRβ. P-INSRβ was the phosphorylated form of INSRβ. The amount of INSRβ was affinity-enriched using the antibody as the method described. Quantitative analysis of the INSRβ phosphorylation using ImageJ software. Error bar was based three biological replicates; 12.5% SDS-PAGE gel. *D*, the variation of expression and phosphorylation levels of INSRβ in the epidermis from sixth instar 24 h larvae to 4-day-old pupae. The antibody against INSRβ was used. ACTB was used as the internal reference; 7.5% SDS-PAGE gel. Statistics were based on the ratio of the density of two bands to the ACTB band using ImageJ software. Statistical analysis was conducted using ANOVA, different letters represented significant differences (*p* < 0.05). The bars indicate the mean ± standard deviations (SD) of three times repetition.

*H. armigera* completes six larval instars, pupae, and adult stages in about 1 month (22). To study the function of INSR, its abundance and phosphorylation profiles during development were examined. Two commercially available antibodies against human non-phospho-INSRβ (INSRβ) and phospho-INSRβ (p-INSRβ) were used for western blotting to detect changes in the INSR protein levels and changes in protein phosphorylation. INSRβ was detected as a single band using the anti-INSRβ antibody in the epidermis, midgut, and fat body. INSRβ levels increased during feeding stages from the sixth instar 6 h to 48 h (sixth-6 h–sixth-48 h) and decreased during metamorphosis from the sixth instar larvae 72 h (sixth-72 h) to the adult (Fig. 1, *A* and *B*). The antibody against INSRβ recognized *H. armigera* INSRβ-His specifically, with a lower molecular band representing the endogenous INSRβ (Fig. S1*A*). The mRNA levels of *Insr* showed similar expression profiles to that of the protein (Fig. S1*B*). These data suggested that INSR expression was reduced during metamorphosis.

To determine the profile of INSRβ phosphorylation, an antibody against p-INSRβ was used after enrichment of same amount of INSRβ to overcome the different expression levels of INSRβ during metamorphosis. Higher levels of p-INSRβ during the larval feeding stages (5F and sixth-6 h–sixth-48 h) compared with that during the metamorphic stages (sixth-72 h–sixth-120 h) were detected (Fig. 1*C*). INSRβ was detected as two bands using the anti-INSRβ antibody and a 7.5% low-concentration gel (Fig. 1*D*). Lambda protein phosphatase (λPPase) treatment decreased the intensity of the p-INSRβ band, suggesting that the upper band was the phosphorylated form of INSRβ (p-INSRβ) (Fig. S1*C*). These results suggested that phosphorylation of INSRβ occurs at a lower level during metamorphosis.

### 20E counteracted insulin-induced INSRβ expression and phosphorylation

**Figure 2:**
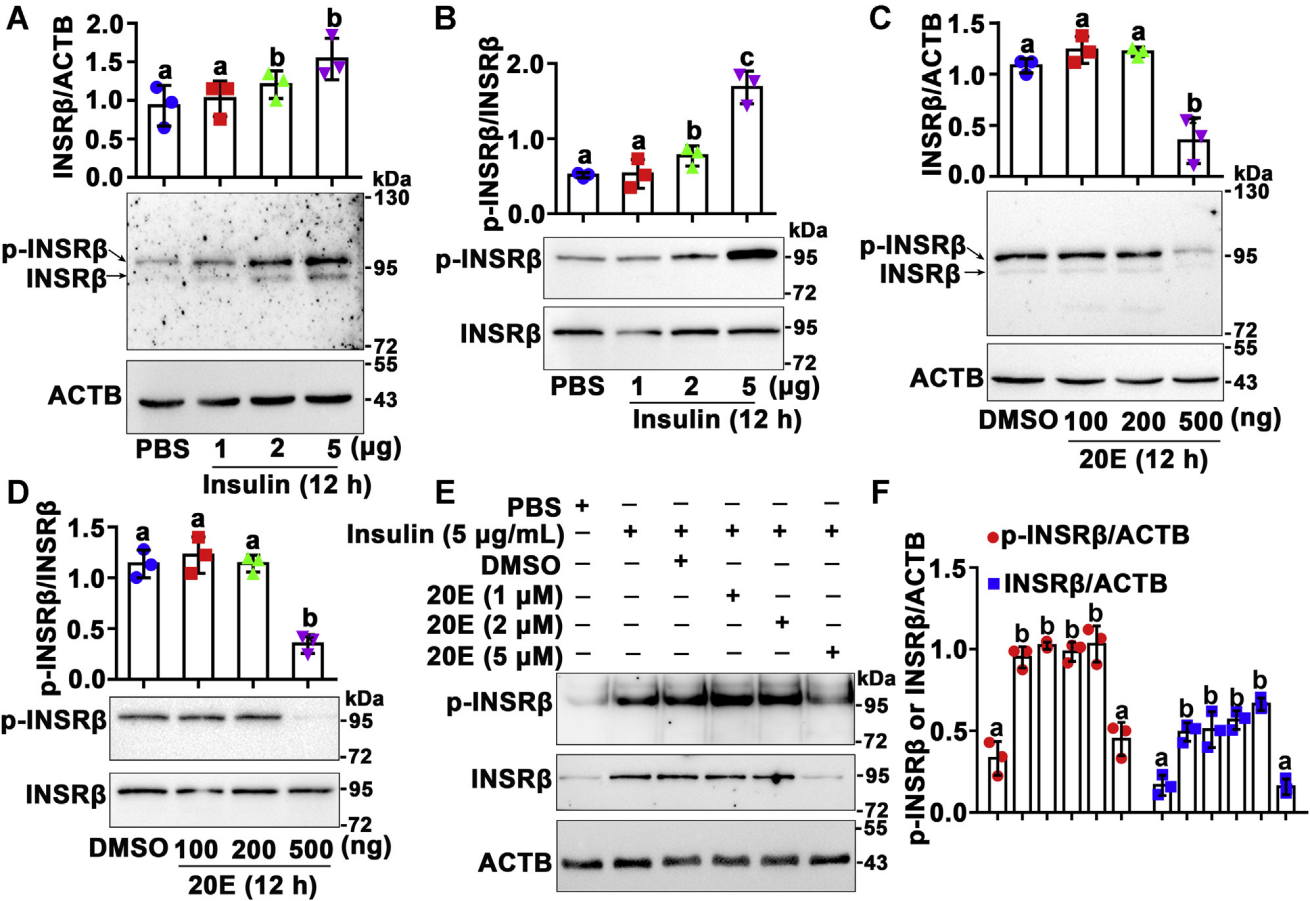
**Insulin and 20E counteractively regulated the abundance and phosphorylation of INSRβ**. In total, 7.5% SDS-PAGE gel for the experiments in addition to indication. *A*, insulin regulation in the expression of INSRβ in the larval epidermis (The purpose of using the epidermis in many places in this article is to keep it consistent with our HaEpi cells). Statistics were based on the ratio of the density of two bands to the ACTB band. The antibody against INSRβ was used. ACTB was used as the internal reference. *B*, insulin regulation on the phosphorylation of INSRβ after affinity enrichment of INSRβ. 12.5% gel. Antibodies against p-INSRβ and INSRβ were used, respectively. *C*, 20E regulation in the abundance of INSRβ in the larval epidermis. Statistics were based on the ratio of the density of two bands to the ACTB band. The antibody against INSRβ was used. ACTB was used as the internal reference. *D*, 20E regulation of the phosphorylation of INSRβ in the larval epidermis after enrichment of INSRβ. 12.5% gel. Antibodies against p-INSRβ and INSRβ were used, respectively. In total, 500 ng 20E was calculated as 5 μM 20E *in vivo* by the formula: c = m/MV, m = 500 ng, M = 480.63 g/mol, V ≈ 200 μl of a sixth-6 h larva. *E*, the effects of overlapped 20E and insulin on INSRβ levels and phosphorylation in HaEpi cells. 12.5% gel. Hormones were added 12 h after starvation (in Grace's medium without FBS). DMSO and PBS were used as solvent controls of 20E and insulin, respectively. The protein amount of each lane was 50 μg. Antibodies against p-INSRβ, INSRβ and ACTB were used, respectively. *F*, statistical analysis of (*E*) using ANOVA, different letters represented significant differences (*p* < 0.05). The statistical analyses of the related density of western blotting bands were performed using ImageJ software based on three independent replicates. The bars indicate the mean ± SD.

The 20E titer *in vivo* appeared to increase from 0.5 μM to 10 μM from larval growth to metamorphosis in *H. armigera* (23, 24). To address the mechanism of the lower level and phosphorylation of INSRβ during metamorphosis, we investigated the roles of insulin and 20E in these events by injecting different concentrations of insulin and 20E into the sixth instar 6 h larvae hemocoel. Increasing concentrations of insulin increased INSRβ levels and phosphorylation in the larval epidermis (Fig. 2*A*). The increased levels of phosphorylated INSRβ were confirmed using the anti-p-INSRβ antibody after normalization of the INSRβ levels by affinity enrichment of INSRβ (Fig. 2*B*). In contrast, a higher concentration of 20E (500 ng) decreased INSRβ levels (Fig. 2*C*) and phosphorylation, detected after affinity enrichment of INSRβ (Fig. 2*D*). In addition, a high concentration of 20E (5 μM) was confirmed to repress insulin-induced INSRβ levels and phosphorylation in HaEpi cells (Fig. 2, *E* and *F*), which represented a model to study INSR in cells. These results suggested that high concentration of 20E represses INSRβ levels and phosphorylation.

### The mechanism by which a high concentration of 20E represses INSRβ phosphorylation

INSRβ undergoes autophosphorylation by binding insulin (14). Therefore, a deficiency of insulin might be the reason for the decrease of INSRβ phosphorylation. However, eight ILPs were identified by BLAST searching of the *H. armigera* genome using the sequences of 38 *Bombyx mori* ILPs, 8 *Drosophila* ILPs (25), and 10 *Homo sapiens* ILPs (Fig. S2), and which ILP binds to INSR was not known; therefore, the expression levels of all *Ilp* genes were detected to address the insulin levels. Surprisingly, the mRNA levels of eight *Ilp* genes increased in the epidermis, midgut, and fat body during metamorphosis from the wandering stage to the later pupal stage, as assessed using quantitative real-time reverse transcription PCR (qRT-PCR) (Fig. S3, *A*–*H*). The *Ilp* genes in the brain also exhibited increased expression during metamorphosis, in addition to *B10-like*, whose relationship with INSRβ requires further study (Fig. S3*I*). Compared with the dimethyl sulfoxide (DMSO) control, 20E-injected in larvae increased their expression of *B2-like*, *B10-like*, and *C1-like* in a dose and time-dependent manner (Fig. S3, *J*–*O*). These findings suggested that the expression levels of most *Ilp* genes are sufficient during metamorphosis and are thus not the reason for the decrease in INSRβ phosphorylation; therefore, INSRβ is likely dephosphorylated by some protein phosphatases under 20E regulation during metamorphosis.

**Figure 3:**
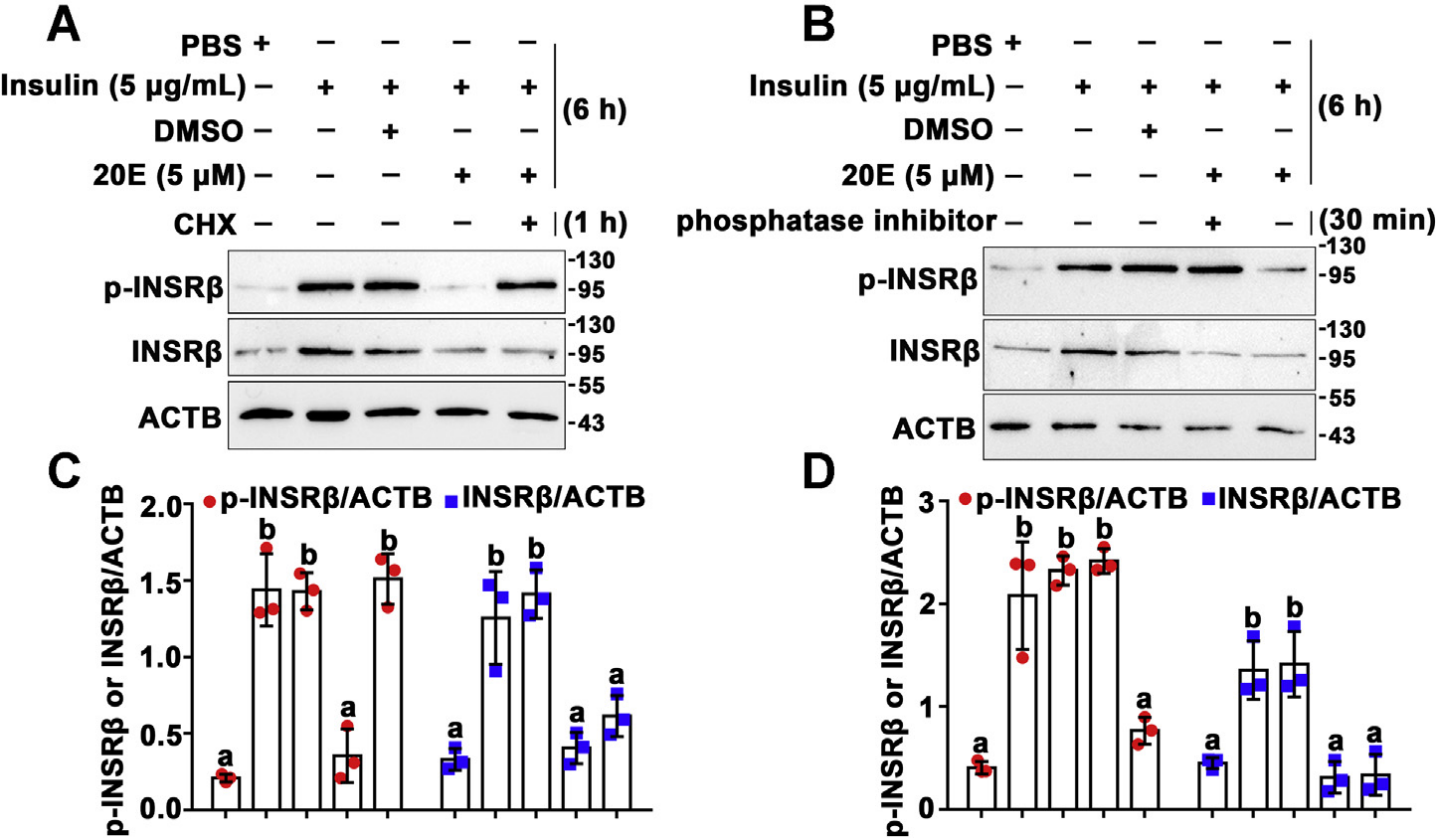
**20E-induced dephosphorylation of INSRβ was repressed by cycloheximide and a phosphatase inhibitor cocktail.***A*, the effect of cycloheximide on 20E-induced dephosphorylation of INSRβ in HaEpi cells (50 μM for 1 h). *B*, the effect of a phosphatase inhibitor cocktail on 20E-induced dephosphorylation of INSRβ in HaEpi cells (1% A and 1% B cocktail for 30 min). 12.5% gel. *C* and *D*, statistical analysis of (*A*) and (*B*) using ANOVA, different letters represented significant differences (*p* < 0.05). The bars indicate the mean ± SD of three times repetition. ImageJ software was used to transform the image data.

The protein synthesis inhibitor, cycloheximide, was used to test the above hypothesis in HaEpi cells. Insulin-induced INSRβ phosphorylation was compared with that in the PBS control. 20E inhibited the insulin-induced INSRβ phosphorylation, compared with that of the DMSO control; however, 20E could not inhibit INSRβ phosphorylation after the cells were preincubated with cycloheximide (Fig. 3, *A* and *C*), suggesting that 20E inhibited INSRβ phosphorylation by promoting the synthesis of certain proteins. Further experiments showed that phosphatase inhibitors (Phosphatase Inhibitor Cocktail, Cat. 20109ES05) repressed 20E-induced INSRβ dephosphorylation (Fig. 3, *B* and *D*), implying that 20E acts *via* a protein phosphatase to dephosphorylate INSRβ.

### PTP1B dephosphorylates INSRβ

**Figure 4:**
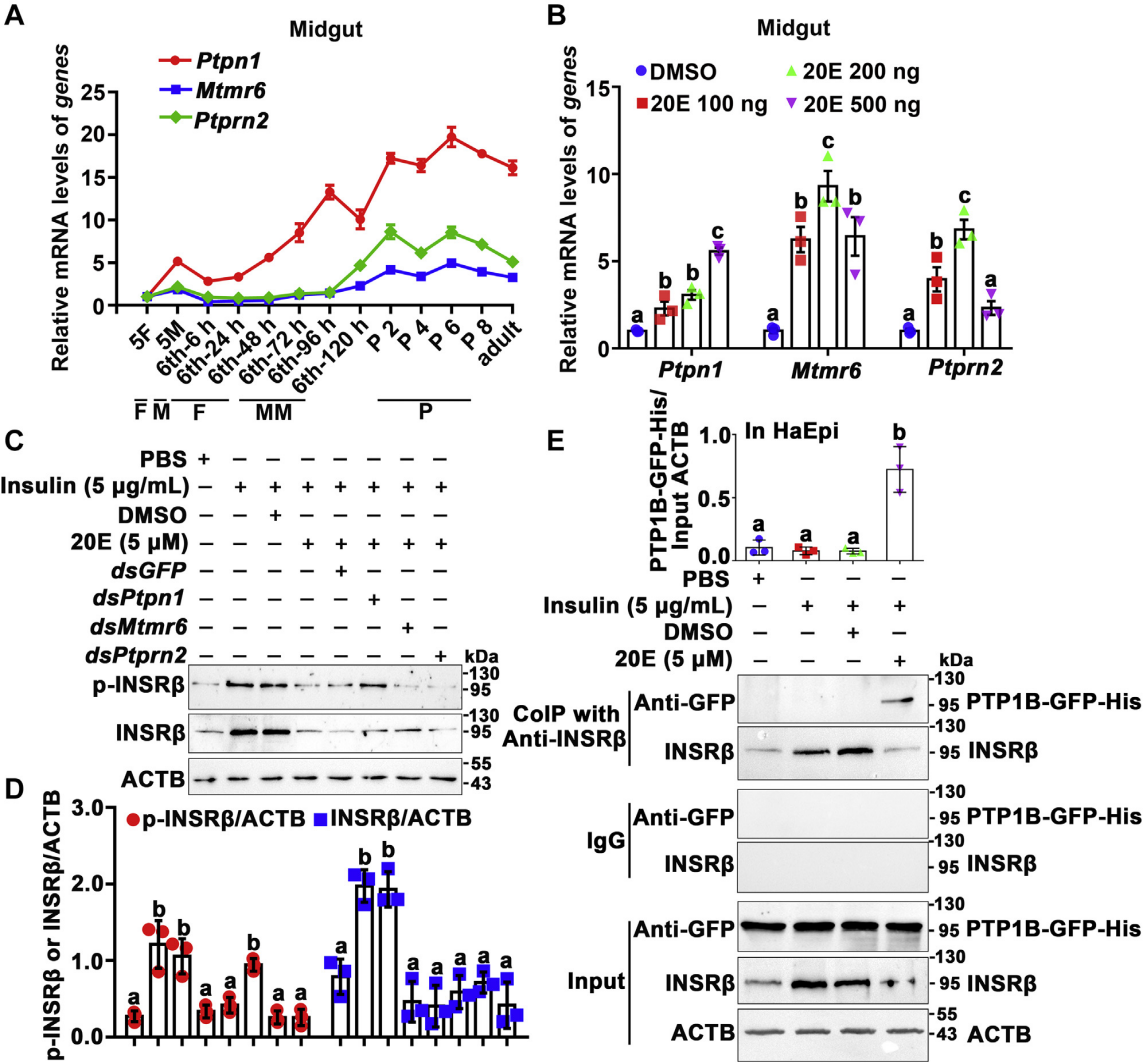
**PTP1B was identified as being involved in 20E-induced dephosphorylation of INSRβ**. *A*, qRT-PCR showing the mRNA expression profiles of *Ptpn1*, *Mtmr6*, and *Ptprn2* in *H. armigera* midgut during development based three repeats. *B*, 20E upregulated the expression of three *PTPases* in the larval midgut. The sixth instar 6 h larvae were injected different concentrations of 20E for 6 h. Equal volume DMSO was used as a control. All the relative mRNA levels were calculated by 2^−ΔΔCT^. The bars indicate the mean ± SD of three times repetition. Statistical analysis was conducted using ANOVA, different letters represented significant differences (*p* < 0.05). *C*, knockdown of *Ptpn1*, INSRβ could not be dephosphorylated by 20E induction. The cells were transfected with 4 μg of *dsGFP* or *dsPTPases* for 48 h. 20E (5 μM) and insulin (5 μg/ml) were added to the cells for 6 h; 12.5% SDS-PAGE. ACTB as the control. *D*, statistical analysis of (*C*) using ANOVA, different letters represented significant differences (*p* < 0.05). The bars indicate the mean ± SD of three replicates. ImageJ software was used to transform the image data. *E*, Co-IP showed the interaction between INSRβ and PTP1B-GFP-His. The cells were treated with PBS, insulin (5 μg/ml), insulin plus DMSO, or insulin plus 5 μM 20E for 6 h after transfected with PTP1B-GFP-His for 48 h. The protein expression levels of INSR, PTP1B-GFP-His, and ACTB in HaEpi cells were detected *via* western blotting following 12.5% SDS-PAGE. INSR was immunoprecipitated with anti-INSRβ, and the coprecipitated PTP1B-GFP-His was detected *via* western blotting analysis using an anti-GFP mAb. Rabbit IgG was used as negative control of the antibody. Statistical analysis was conducted using ANOVA, different letters represented significant differences (*p* < 0.05). The bars indicate the mean ± SD of three replicates. ImageJ software was used to transform the image data.

To determine the phosphatase that phosphorylates INSRβ, we identified three highly expressed protein-tyrosine-phosphatase (PTPase) genes, *Ptpn1*, *Mtmr6*, and *Ptprn2*, during metamorphosis from the midgut transcriptomes at the sixth-24 h feeding stage and sixth-72 h metamorphic stage, using the Illumina sequencing platform, a database produced in our laboratory. The transcriptome had only been determined once; therefore, the phosphatases were further examined for their developmental expression profiles in tissues using qRT-PCR. The transcript levels of the three *PTPases* increased in the midgut during the metamorphic stages (sixth-72 h to adult) compared with that in the feeding stages (sixth-6 h–sixth-48 h) (Fig. 4*A*), and 20E increased their expression (Fig. 4*B*), suggesting that they might be involved in 20E-induced INSRβ dephosphorylation.

To identify which of the three PTPases dephosphorylates INSRβ, we knocked down their expression in HaEpi cells using RNAi, separately. Insulin induced INSRβ phosphorylation, compared with PBS, and 20E decreased insulin-induced INSRβ phosphorylation compared with DMSO. However, knockdown of *Ptpn1* maintained INSRβ phosphorylation, whereas after knockdown of *Mtmr6* and *Ptprn2*, 20E still induced INSRβ dephosphorylation, compared with *dsGFP* (Fig. 4, *C* and *D*). The efficacy of *PTPase* knockdown was demonstrated using qRT-PCR analysis (Fig. S4). Coimmunoprecipitation (Co-IP) experiments using the antibody against INSRβ (anti-INSRβ) confirmed that the overexpressed PTP1B-GFP-His protein interacted with INSRβ under 20E induction (Fig. 4*E*). These data suggested that PTP1B dephosphorylates INSRβ.

### PTEN is involved in INSRβ dephosphorylation by regulating FoxO nuclear localization to upregulate PTP1B expression and repress INSRβ expression

**Figure 5:**
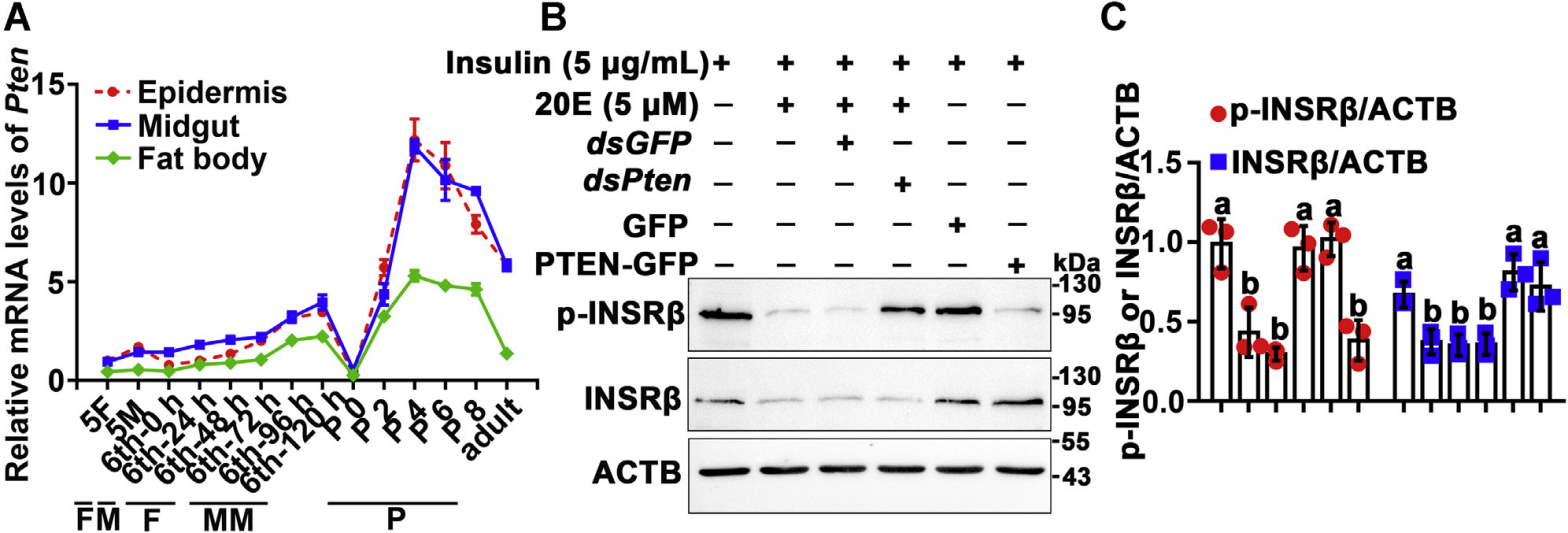
**PTEN is involved in 20E-induced dephosphorylation of INSRβ**. *A*, qRT-PCR showing the expression profiles of *Pten* in various tissues during development, with *Actb* as the quantity and quality control. *B*, western blotting showing the effect of knockdown of *Pten* or overexpression of *Pten* in HaEpi cells on INSRβ; 12.5% SDS-PAGE. *C*, statistical analysis of (*B*) using ANOVA, different letters represented significant differences (*p* < 0.05). The bars indicate the mean ± SD of three times repetition. ImageJ software was used to transform the image data.

PTEN is a dual-function phosphatase playing roles in the cell membrane (26), and its expression is upregulated by 20E during metamorphosis (20); therefore, its involvement in INSRβ dephosphorylation was examined. The mRNA levels of *Pten* increased during metamorphosis from the wandering stage to the pupal stage in the epidermis, midgut, and fat body (Fig. 5*A*). A 20E receptor EcR-binding element (EcRE) 5′-AATGGCAATGACTAC-3′ (−1060 to −1074 bp, relative to ATG) was predicted (http://jaspardev.genereg.net/) in the promoter region of *Pten*. 20E increased the transcript level of *Pten* in a dose- and time-dependent manner, compared with that in the DMSO control (Fig. S5, *A* and *B*). In HaEpi cells, 20E reduced the insulin induced-p-INSRβ levels; however, knockdown of *Pten* blocked 20E-induced dephosphorylation of INSRβ, compared with *dsGFP*. In contrast, overexpression of *Pten* decreased INSRβ phosphorylation in the absence of 20E induction, compared with that in cells overexpressing green fluorescent protein (GFP) (Fig. 5*B*). The statistical analysis confirmed the data (Fig. 5*C*). The efficacy of *Pten* knockdown was demonstrated using qRT-PCR analysis (Fig. S5*C*), and the overexpression of *Pten* was indicated using western blotting (Fig. S5*D*). However, Co-IP experiments did not identify an interaction between INSRβ and PTEN (Fig. S5*E*). These results indicated that PTEN is involved in INSRβ dephosphorylation, but it cannot interact with INSRβ directly.

**Figure 6:**
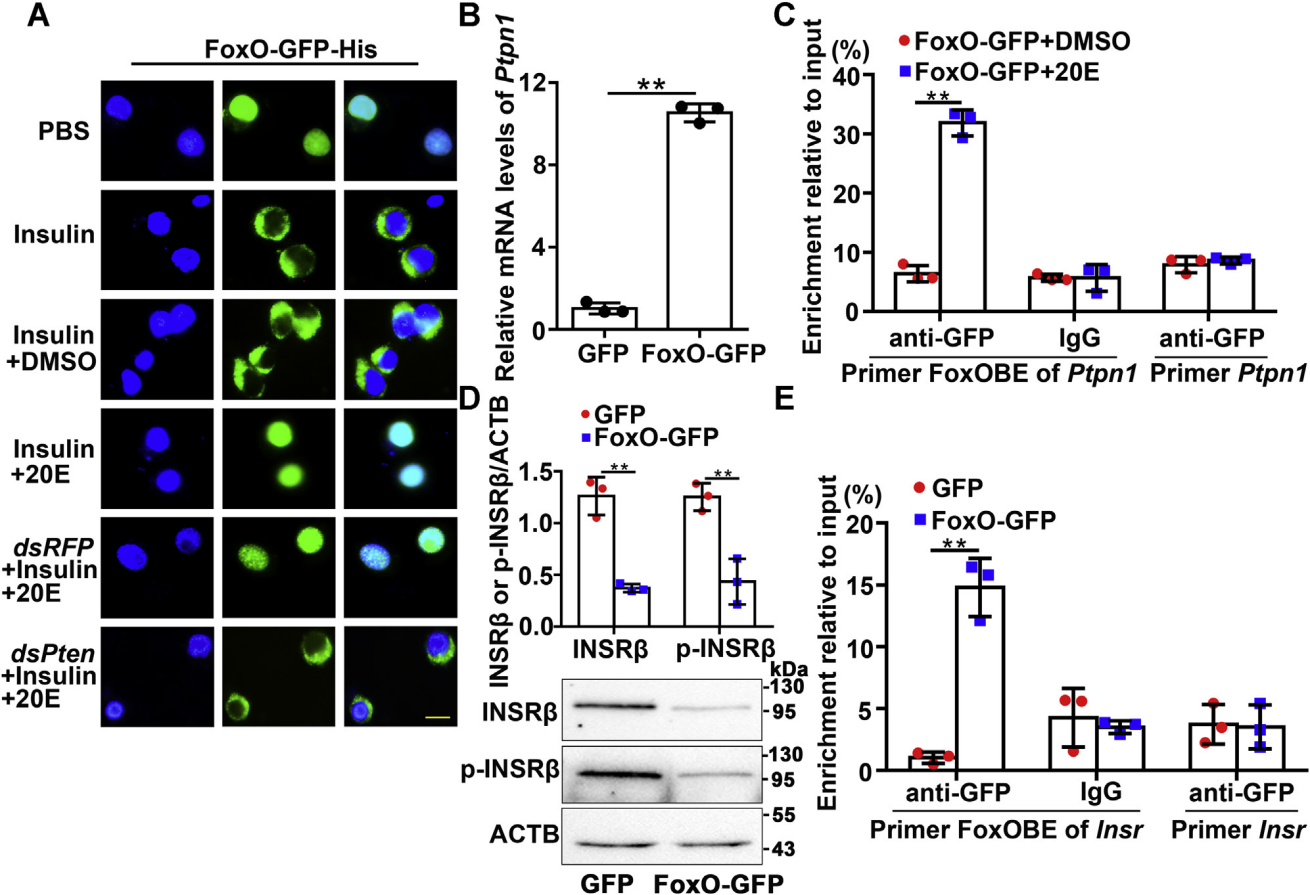
**PTEN determined FoxO nuclear localization and its opposite transcriptional functions in the 20E and insulin pathway.***A*, the subcellular localization of FoxO-GFP-His in the cells in various conditions (insulin 5 μg/ml, 20E 5 μM for 6 h) without FBS. *Green*, *green* fluorescence from FoxO-GFP. *Blue*, nucleus stained with 4′-6-diamidino-2-phenylindole dihydrochloride (DAPI). Scale bars: 20 μm. *B*, qRT-PCR showing the increased *Ptpn1* expression levels after overexpression of FoxO-GFP in HaEpi cells. *C*, ChIP analysis of 20E-induced FoxO binding to the FoxOBE in the *Ptpn1* promoter region. FoxO-GFP-His was overexpressed in HaEpi cells for 48 h followed by inducing of 5 μM 20E or DMSO for 6 h. Input: nonimmunoprecipitated chromatin. IgG, nonspecific rabbit IgG. Primer FoxOBE of *Ptpn1*: primers targeted the *Ptpn1* FoxOBE-containing sequence. Primer *Ptpn1*: primers targeted to *Ptpn1* ORF. *D*, western blotting showing the repression of INSRβ expression and phosphorylation by the overexpression of FoxO-GFP; 12.5% SDS-PAGE gel. Antibodies against INSRβ and p-INSRβ were used to detect the INSRβ expression and phosphorylation levels. All statistical analyses of the density of western blotting bands were performed with ImageJ software. *E*, ChIP analysis of FoxO binding to the FoxOBE in the *Insr* promoter region. FoxO-GFP-His or GFP-His was overexpressed in HaEpi cells for 48 h. Input: nonimmunoprecipitated chromatin. IgG, nonspecific rabbit IgG. Primer FoxOBE of *Insr*: primers targeted the *Insr* FoxOBE-containing sequence. Primer *Insr*: primers targeted to *Insr* ORF. All the experiments were performed in triplicate, and statistical analysis was conducted using Student’s *t*-test (∗∗*p* < 0.01). The bars indicate mean ± SD.

A previous study showed that PTEN induces FoxO nuclear localization by repressing AKT and FoxO phosphorylation in *H. armigera* under 20E induction (20); therefore, PTEN was suspected to play roles in 20E-induced INSRβ dephosphorylation by regulating FoxO nuclear localization, thereby promoting *Ptpn1* expression. Therefore, FoxO fused with a GFP tag was overexpressed in HaEpi cells to address the mechanism of PTEN’s involvement in 20E-induced INSRβ dephosphorylation, with GFP showing the subcellular location of FoxO. FoxO-GFP and GFP were confirmed as overexpressed in HaEpi cells, separately (Fig. S6*A*). The overexpressed FoxO-GFP was localized in the nucleus in the PBS control and translocated into the cytoplasm after insulin induction. Compared with the insulin plus DMSO control, 20E reversed insulin’s function and maintained FoxO-GFP’s nuclear localization. However, compared with *dsRFP* plus insulin and 20E, after knockdown of *Pten*, 20E could not maintain FoxO-GFP’s nuclear localization (Fig. 6*A*). The control group overexpressing the GFP tag did not show the subcellular variation in FoxO localization (Fig. S6*B*), confirming that PTEN determines FoxO-GFP nuclear localization.

The effect of FoxO-GFP nuclear localization on the expression of PTP1B was examined to address its role in PTP1B expression. A FoxO-binding element (FoxOBE) 5′-TTGTTAAC-3′ (−980 to −973 bp, relative to ATG) was predicted in the promoter region of *Ptpn1*, which was similar to the FoxOBE sequence (5′-TTGTTTAC-3′) in the promoter region of *H. armigera* BrZ7 (20). In the FoxO-GFP overexpressing cells, the *Ptpn1* level increased compared with that in the GFP overexpressing cells (Fig. 6*B*), suggesting that the overexpressed FoxO-GFP enhanced *Ptpn1* expression. A chromatin immunoprecipitation (ChIP) assay showed that FoxO-GFP bound more FoxOBE under 20E treatment than it did in the DMSO treatment control using the primer FoxOBE of *Ptpn1* (FoxOBE-*Ptpn1*-F/FoxOBE-*Ptpn1*-R primers targeting the *Ptpn1* FoxOBE-containing sequence, as shown in the Table S1). The IgG negative control and *Ptpn1* primers (Ptpn1-F/R located in the open reading frame (ORF) of *Ptpn1*) did not produce the same results (Fig. 6*C*). These results suggested that 20E *via* FoxO promotes *Ptpn1* transcription.

Next, the effect of FoxO-GFP nuclear localization on the level of INSRβ was examined because another kind of FoxOBE, 5′-TGTTTAC-3′ (−1835 to −1829 bp, relative to ATG), was predicted in the promoter region of *Insr*. Interestingly, in the FoxO-GFP overexpressing cells, the protein level of INSRβ decreased, in addition to decrease of INSRβ phosphorylation by PTP1B (Fig. 6*D*), suggesting that overexpression of FoxO-GFP repressed *Insr* expression. ChIP results further showed that FoxO-GFP bound more FoxOBE than GFP, using the primer FoxOBE of *Insr* (FoxOBE-*Insr*-F/FoxOBE-*Insr*-R primers targeting the *Insr* FoxOBE containing sequence, as shown in the Table S1). The IgG negative control and *Insr* primers (Insr-F/R, located in the open reading frame of *Insr* Table S1) did not produce the same results (Fig. 6*E*). These findings suggested that FoxO is the repressor of *Insr* transcription.

### Knockdown of *Ptpn1* in larvae maintained INSRβ phosphorylation and led to small pupae and earlier pupation by increasing the 20E titer

**Figure 7:**
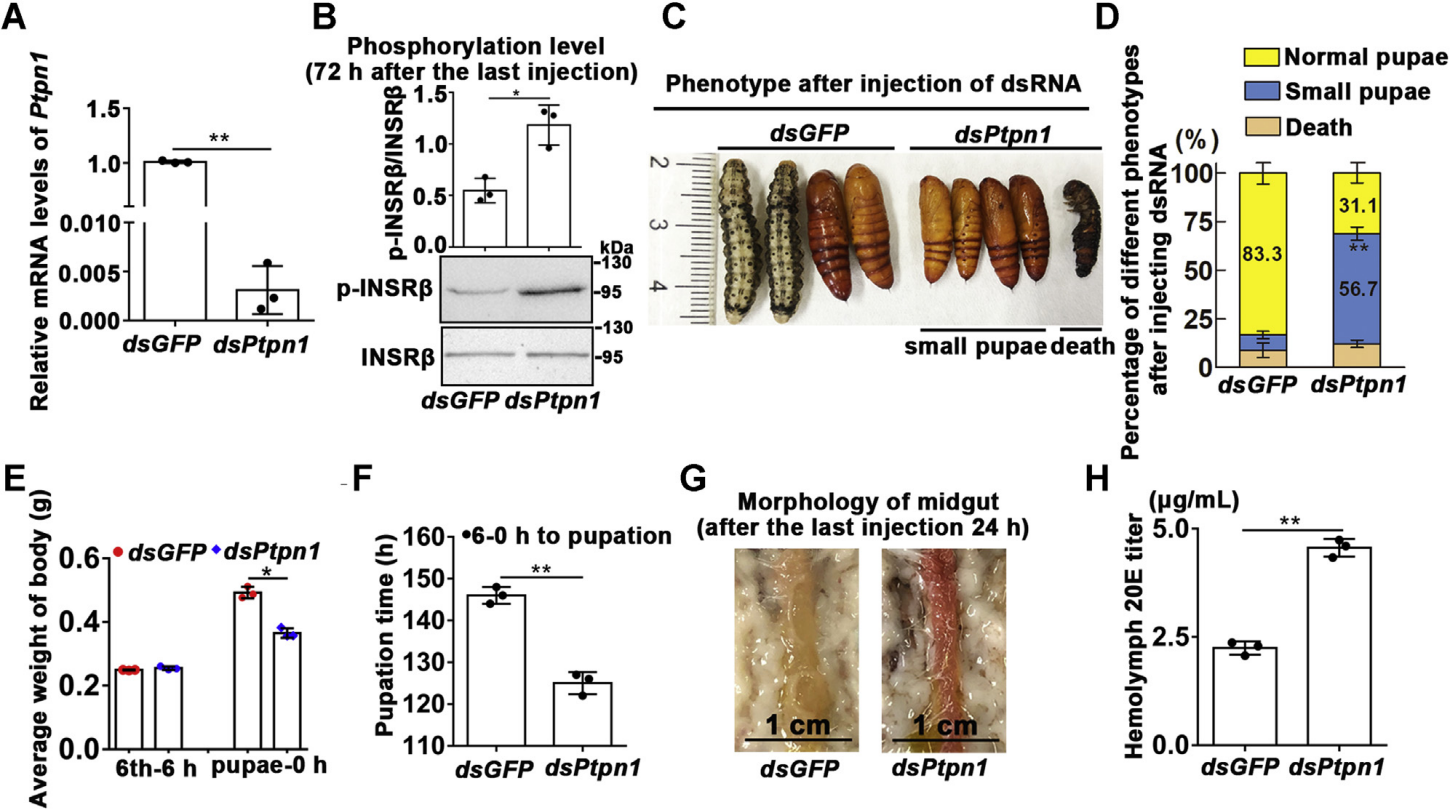
***Ptpn1* knockdown *via* RNAi in larvae accelerated pupation time, formed small pupae, and increased the 20E titer.***A*, efficacy of *Ptpn1* knockdown in the epidermis of larvae by qRT-PCR analysis (sixth instar 6 h larva for the first *dsPtpn1* or *dsGFP* injection, 24 h later for the second injection, 1.5 μg dsRNA/larva each time). *B*, western blotting showed the maintained phosphorylation of INSRβ 72 h after the last injection of *dsPtpn1*, compared with the *dsGFP* at the same time. INSRβ and p-INSRβ antibodies were used, respectively; 12.5% SDS-PAGE gel. ImageJ software was used to transform the image data. *C*, phenotypes after *Ptpn1* knockdown observed from 114 h to 138 h after first dsRNA injection. *D*, the statistical analysis of percentage of different phenotypes based the experimental group and control group. Each group contains 30 larvae. The statistical analysis was performed using three independent replicates by Student’s *t*-test. *E* and *F*, statistical analysis of pupation weight and pupation time. *G*, midgut morphology observed after the last injection 24 h. *H*, the 20E titer increased in hemolymph 66 h after the last injection of *dsPtpn1*, compared with the *dsGFP* at the same time. The statistical analysis was performed using three times repetition by Student’s *t*-test (∗*p* < 0.05; ∗∗*p* < 0.01). The bars indicate the mean ± SD.

To examine the function of PTP1B *in vivo*, we knocked down *Ptpn1* by injecting *dsPtpn1* into the sixth instar 6 h larval hemocoel. qRT-PCR showed that the expression of *Ptpn1* was successfully knocked down in the epidermis (Fig. 7*A*). The level of phosphorylated INSRβ after injection of *dsPtpn1* was significantly higher than that in the *dsGFP* control group (Fig. 7*B*), which confirmed the role of PTP1B in INSRβ dephosphorylation *in vivo*. As a consequence, the major effects of knockdown of *Ptpn1* were the production of smaller pupae (Fig. 7, *C*–*E*) and earlier pupation, compared with that in the control group (Fig. 7*F*). In addition, the larval midgut appeared red, which normally occurs during larval midgut programmed cell death (PCD) during metamorphosis (27, 28), in the *dsPtpn1* group, but not in the *dsGFP*-treated larval midgut at the same time after injection of double-stranded RNA (dsRNA) (Fig. 7*G*). This suggested that the larvae of *dsPtpn1* entered metamorphosis earlier than the control group. The 20E titer in the hemolymph was 5 μM, which was higher than that (2.5 μM) in the *dsGFP* control (Fig. 7*H*), indicating that maintaining phosphorylation of INSRβ increased 20E production, which accelerated pupation.

### Knockdown of *Insr* in larvae led to small pupae and delayed pupation and increased hemolymph glucose levels by decreasing the 20E titer

**Figure 8:**
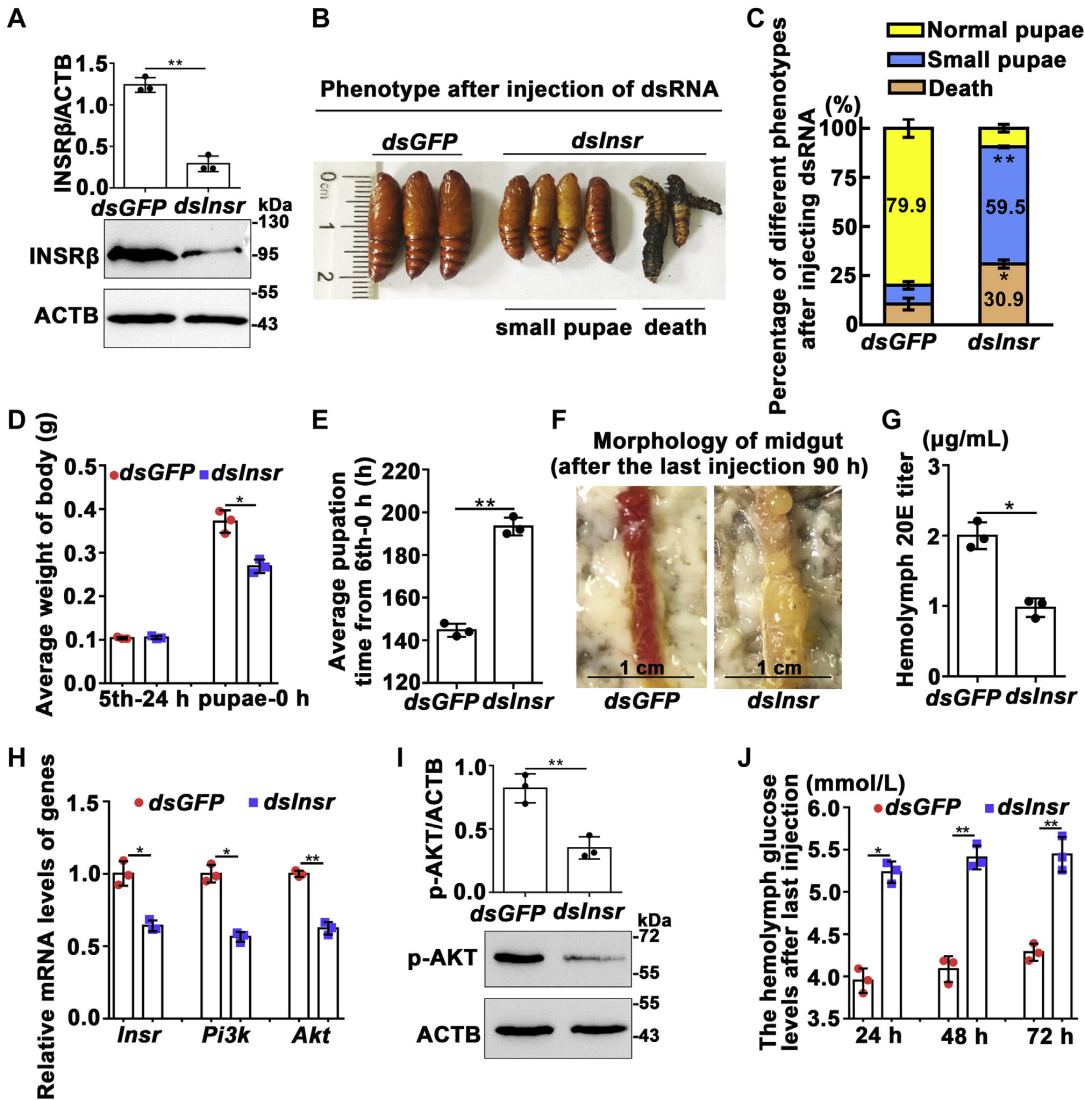
**Knockdown of *Insr* in fifth instar larvae *via* dsRNA injection delayed pupation time, produced small pupae, and decreased the 20E titer.***A*, Western blotting showing the efficacy of *Insr* knockdown. The larvae epidermis protein was extracted after the last injection 24 h; 12.5% gel. ImageJ software was used to transform the image data. *B*, insect phenotypes after three times injection of *dsInsr* or *dsGFP* into the larval hemocoel (1.5 μg/larva, in fifth instar 24 h, 48 h, and in sixth instar 6 h). Phenotypes were observed from 90 h to 144 h after the third injection. *C*, the statistical analysis of different phenotypes based the experimental group and control group. Each group contains 30 larvae with three independent repeats. % was calculated by the number of phenotypes in the 30 samples. Error bars indicate the mean ± SD of three repeats. Student’s *t*-test was performed to analyze the difference of the two groups of *dsGFP* and *dsInsr*. *D* and *E*, statistical analysis of body weight and pupation time. *F*, midgut morphology observed 90 h after the last injection. *G*, the 20E titer detection in the hemolymph after the last injection of dsRNA for 90 h. *H*, qRT-PCR detected the mRNA levels of *Insr*, *Pi3k*, and *Akt* after the last injection of dsRNA for 24 h. *I*, *Insr* knockdown decreased AKT phosphorylation after the last injection of dsRNA for 24 h. The antibody against p-AKT was used. *J*, the hemolymph glucose levels detection after the last injection of dsRNA for 24 h, 48 h, 72 h. The statistical analyses were conducted using Student’s *t*-test (∗*p* < 0.05; ∗∗*p* < 0.01) based triplicate. Error bars indicate the mean ± SD.

To examine the function of INSR in insect growth and insulin function, we injected *dsInsr* into fifth instar 24 h larvae to knock down *Insr*. Western blotting showed that the level of the INSRβ protein was successfully reduced (Fig. 8*A*). This resulted in 30.9% death among the fifth instar and the sixth instar larvae and 59.5% small pupae (Fig. 8, *B* and *C*). Moreover, the pupal weight of the *dsInsr*-treated group decreased to an average of 0.26 g compared with 0.37 g of the *dsGFP* injection control (Fig. 8*D*). The pupation time was delayed significantly from 140 h to 190 h after knockdown of *Insr* (Fig. 8*E*), and the midgut appeared red later than that in the *dsGFP* control (Fig. 8*F*), suggesting that INSR plays roles in larval growth and pupation. The reason for the delayed pupation might because of insufficient 20E in the hemolymph (Fig. 8*G*) along with the delayed larval growth when *Insr* was knocked down. Meanwhile, the mRNA levels of insulin pathway genes- *Insr*, *Pi3k*, and *Akt* were downregulated after *Insr* knockdown (Fig. 8*H*), and western blotting analysis showed that AKT phosphorylation also decreased after *Insr* knockdown (Fig. 8*I*), suggesting that knockdown of *Insr* blocked insulin signaling. The hemolymph glucose levels in *dsInsr*-injected larvae were significantly higher than those of *dsGFP*-injected larvae (Fig. 8*J*). These results suggested that INSR functions in larval growth, 20E production, hemolymph glucose metabolism, and metamorphosis.

### 20E increased hemolymph glucose levels during metamorphosis

**Figure 9:**
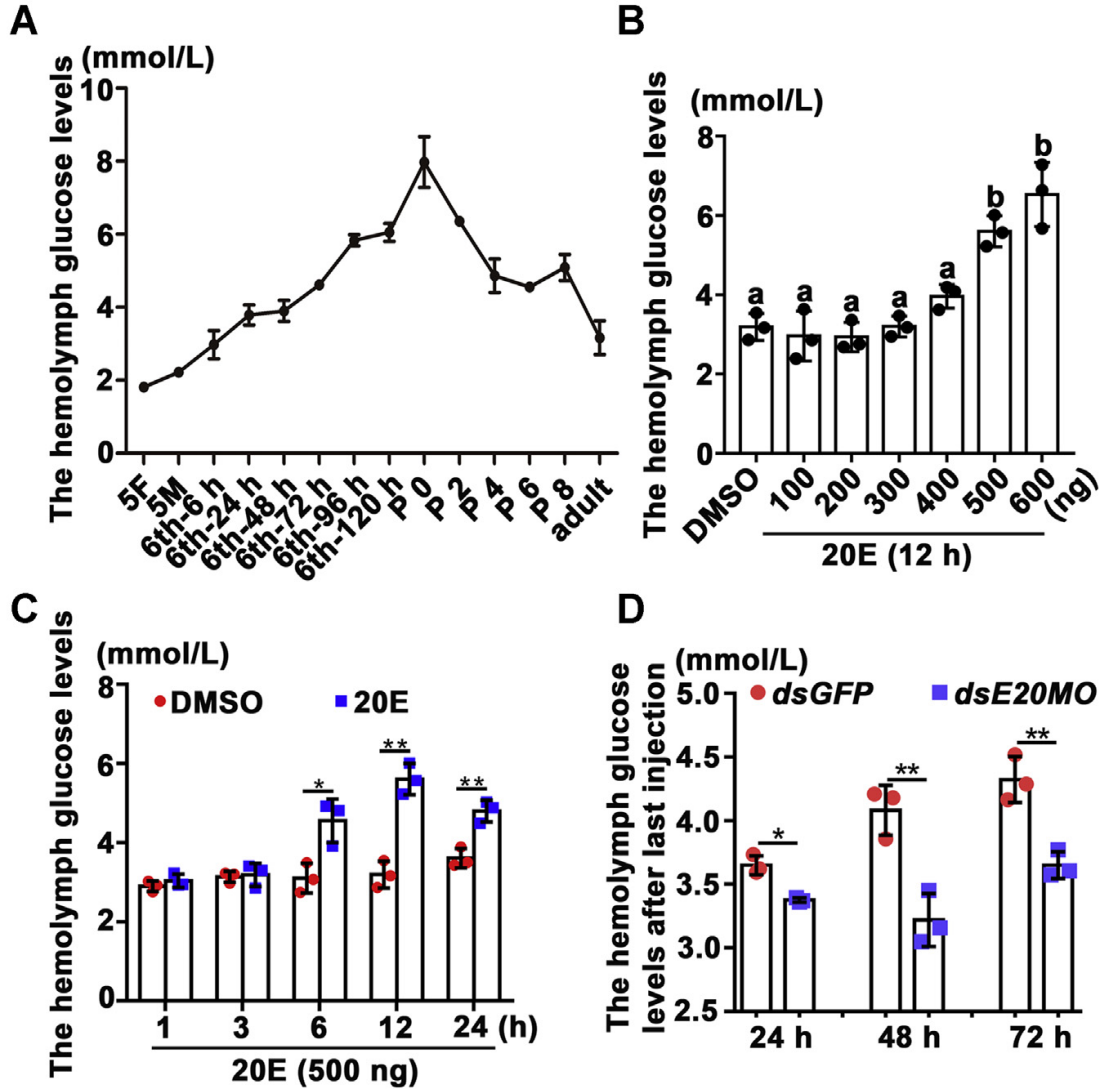
**20E increased hemolymph glucose levels during metamorphosis.***A*, the hemolymph glucose levels during development. The error bars were calculated from three independent repeats. *B* and *C*, the increase of the hemolymph glucose levels by 20E induction in dose and time. DMSO was used as the solvent control. *D*, the hemolymph glucose levels detection after the last injection of dsRNA for 24 h, 48 h, 72 h. Statistical analysis was conducted using ANOVA for (*B*), different letters represented significant differences (*p* < 0.05). Statistical analysis of the data in *C* and *D* by Student’s *t*-test from three biological repeats (∗*p* < 0.05; ∗∗*p* < 0.01). The bars indicate the mean ± SD of three times repetition.

We hypothesized that hemolymph glucose levels would be high because of the decrease in INSRβ expression and phosphorylation during metamorphosis; therefore, the hemolymph glucose levels were detected. The hemolymph glucose levels increased until pupation, even when the larvae had stopped feeding from sixth 72 h, and then declined gradually to the adult stage (Fig. 9*A*), suggesting that the hemolymph glucose levels not only accumulated along with INSR insufficiency but also were increased by gluconeogenesis by high levels of 20E during metamorphosis. Injection of 500 ng (equivalent to about 5 μM *in vivo*) or higher concentrations of 20E in 6th instar 6 h larvae increased hemolymph glucose levels compared with those in the DMSO control (Fig. 9, *B* and *C*). To verify that the increase of the hemolymph glucose was induced by 20E, we injected *dsE20MO* into fifth instar 24 h larvae to knock down the gene encoding ecdysone 20-monooxygenase (E20MO), an enzyme that converts ecdysone (E) to the active 20E in insects (29). The hemolymph glucose levels decreased significantly in *dsE20MO*-injected larvae (Fig. 9*D*). The efficacy of *E20MO* knockdown by RNAi in epidermis was confirmed using qRT-PCR (Fig. S7). The results suggested that high concentrations of 20E cause high hemolymph glucose levels during metamorphosis.

## Discussion

In the present study, we revealed that INSR plays a significant role in insulin pathway to promote insect larval growth, thereby producing more steroid hormone 20E during larval feeding stages. The increased 20E promotes PTEN expression, which localizes FoxO in the nucleus to promote PTP1B expression and repress INSR expression. PTP1B dephosphorylates INSRβ to block the insulin pathway, resulting in cessation of larval growth. In the hemolymph, glucose accumulates by blocking of the insulin pathway. These findings revealed one of the mechanisms by which 20E counteracts insulin to stop larval growth and cause glucose accumulation in the hemolymph.

### The critical 20E titer blocks the insulin pathway, thereby stopping larval growth and determining body size

Body size control is determined by various factors (2), including nutrition (18), insulin, and steroid hormones (3). In *Drosophila* (30) and *Manduca* (31), larvae must surpass the critical weight for metamorphosis. The critical weight is achieved during 8 to 12 h after ecdysis to the third instar (20 h earlier than the wandering stage) in *Drosophila* (18). The INSR signaling pathway plays an indispensable role in growth and metabolism (32, 33). Our study showed that knockdown of *Ptpn1* accelerated pupation time and produced small pupae because INSR phosphorylation was maintained and the 20E titer reached the critical titer earlier than in the control. However, knockdown of *Insr* delayed pupation and produced small pupae because of slower growth and an insufficient 20E titer, although the effects might be caused by the delayed processes. Taken together, the INSR functions in the insulin pathway to ensure that larvae grow to reach the critical weight to produce the critical 20E titer during larval feeding stages, which counteracts the insulin pathway and triggers metamorphosis to determine body size. The increase of 20E after larval feeding cessation probably comes from the gonads (34, 35).

The 20E titers vary *in vivo* from larval growth to metamorphosis (36): 1 to 4 μg/ml (2–9 μM) in *Manduca sexta* (37), 0.5 to 5 μg/ml (1–10 μM) in the hemolymph of *Antheraea mylitta* (38), and 0.2 to 6 μM in the whole body (24) and 0.5 to 10 μM in hemolymph of *H. armigera* (23). Several studies showed that 20E functions to promote cell proliferation at low concentrations in *M. sexta* (39), but promotes apoptosis at high concentrations in *H. armigera* (40). Low concentrations of 20E stimulate the rapid proliferation of cells in wing discs in *B. mori* (41), while high concentrations of 20E inhibit the proliferation of wing discs and stop the growth of surrounding tissues (42). The mechanisms include the fact that 20E increases the cellular calcium level in a concentration-dependent manner (43), which induces autophagy or apoptosis according to the calcium concentration, respectively (44).

### 20E increases glucose levels and *Ilp* expression

20E upregulates insulin-like peptide 6 (DILP6) expression for postfeeding growth in *Drosophila* (45). Here we also detected increased expression of *Ilp* genes during metamorphosis under 20E regulation. 20E competes with dopamine to bind to the dopamine receptor (DopEcR) to stop the larval feeding behavior during metamorphosis (24); therefore, the *Ilp* genes were not induced by food ingestion during metamorphosis. Glucose has been found to promote insulin production in humans (46) and in *Drosophila* (47). Whether the increased glucose in the hemolymph during metamorphosis induces the expression of *Ilp* genes requires further study. The compensatory increase in ligands is another possibility when the receptors are insufficient (48). Bombyxin, the first ILP found in *B. mori*, promotes wing disc development (49). Our study suggested that the different ILPs might promote larval or adult tissue growth by binding INSR or other receptors.

Surprisingly, 20E increased hemolymph glucose levels during metamorphosis when *Ilp* expression was sufficient, although the protein levels of ILPs should be examined in a future study. The high hemolymph glucose levels might occur because glucose cannot be taken up into cells, synthesized to glycogen, or utilized to produce energy under conditions of insufficient INSR expression and phosphorylation during metamorphosis under 20E regulation. The continuously increased hemolymph glucose levels during metamorphosis after larvae stopped feeding suggested the presence of active glycogenolysis and gluconeogenesis under repression of glucose utilization by 20E induction. These hypotheses require clarification in a future study.

The abnormal glucose increase with sufficient insulin levels in the blood is the typical characteristic of type 2 diabetes (T2D) in humans; however, the mechanisms are unclear (50). A fasting plasma glucose level ≥7.0 (mmol/l) is the clinical indication of diabetes in humans according to the World Health Organization (51). Our study suggested that high levels of the steroid hormone 20E increase hemolymph glucose levels by repressing INSR expression and phosphorylation. The 20E-induced increase in hemolymph glucose in *H. armigera* was less than 7 mmol/l but did reach 8 mmol/l in day 0 pupae during metamorphosis; therefore, the high levels of hemolymph glucose should have physiological significance for imaginal organ development after feeding cessation.

In mammals, glucocorticoids can inhibit INSR phosphorylation in rat skeletal muscle (52). The glucocorticoid analog dexamethasone inhibits insulin signaling and suppresses phosphorylation of INSR (53). PTP1B plays roles in dexamethasone action (54). Total INSR levels are also reduced by dexamethasone (55). *Drosophila* is used to study diabetes because it has a very similar insulin pathway to that of humans (47). *H. armigera* represents another model to study steroid-induced hemolymph glucose regulation and the relationship between larval tissue PCD and adult tissue formation.

### FoxO represses INSR expression

The transcriptional regulation of insulin pathway genes remains unclear because the positive transcription factor of the insulin pathway is unknown. In mammals and *D. melanogaster*, FoxO1 activation leads to an increase in INSR protein levels through a feedback mechanism under conditions of low nutrient levels (56). However, in *H. armigera*, FoxO shows increased expression during metamorphosis and is localized in the nucleus where it exerts a transcription factor role under 20E regulation (20). In contrast, INSR expression is decreased during metamorphosis under 20E regulation; therefore, FoxO is not able to increase INSR expression in *H. armigera*. A FoxOBE motif was found in the 5′ upstream region of *Insr*, and ChIP experiments confirmed that FoxO could bind to it; however, the overexpression of FoxO repressed *Insr* expression. Our study revealed that FoxO functions as a transcriptional repressor of *Insr*, representing one of the mechanisms of transcriptional regulation of the insulin pathway genes *via* FoxO’s subcellular localization. The different results among these studies imply that the roles of FoxO differ under different conditions or species. In addition, the contributions of other cofactors involved in the binding of FoxO to FoxOBE require further study.

### The insulin pathway is conserved in insects and humans

Phylogenetic analysis showed a short evolutionary distance of INSR from *H. armigera* (XP_021192467. 1) to that of other insect species (Fig. S8*A*). Three potential tyrosine phosphorylation sites (Tyr1146, Tyr1150, and Tyr1151) were completely conserved from insects to mammals (Fig. S8*B*). Although insulin is secreted by pancreatic B cells in mammals (57), and insect ILPs are produced in various tissues, including the brain (1), the function of ILPs and INSRs are conserved from insects to humans (49). Mammal insulin could induce the insect insulin pathway in *H. armigera* (58). These data suggested the conservation of function of the insulin pathway in insects and mammals.

## Conclusion

**Figure 10:**
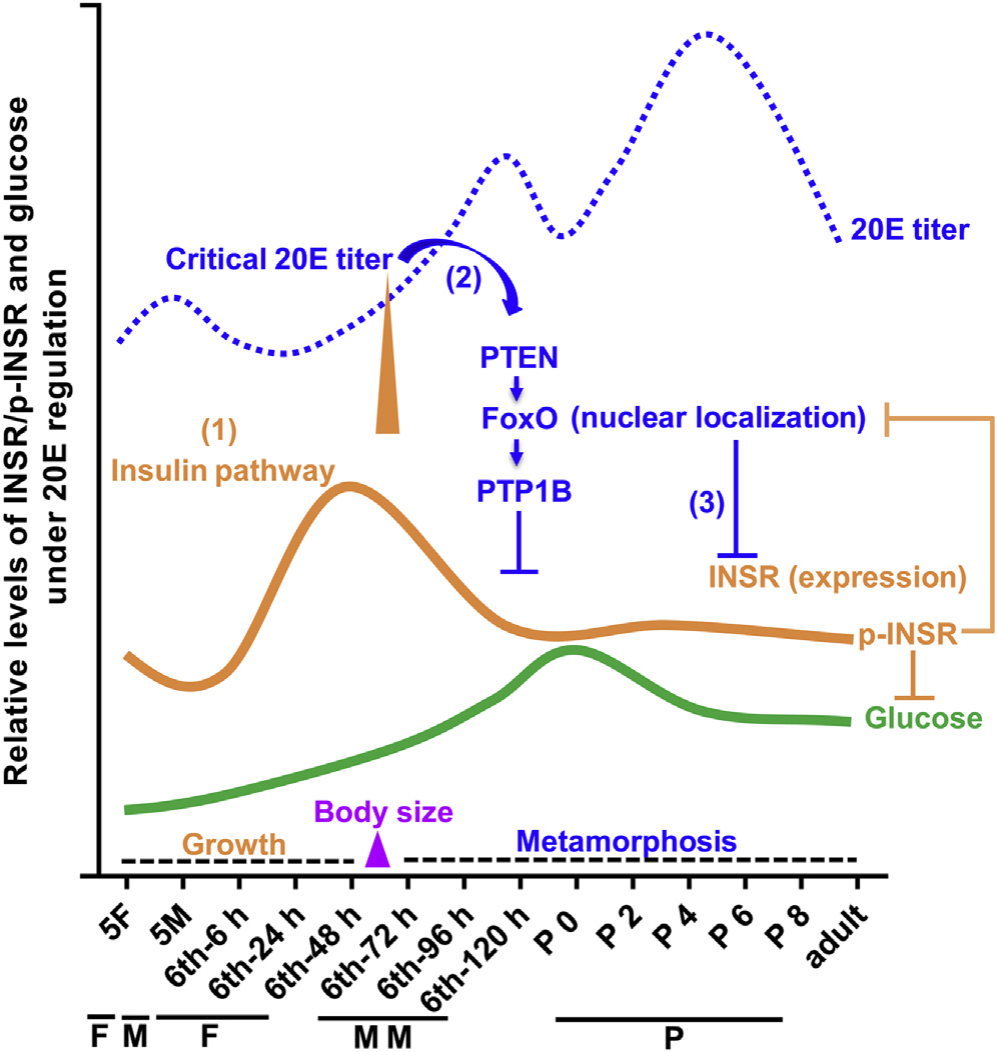
**Chart explaining the possible mechanism of 20E counteracting insulin pathway to stop larval growth and determine body size.** The 20E titer increased during metamorphosis in *H. armigera* (23, 24). INSR played roles to promote insect larval growth and 20E production to the critical titer by repressing FoxO nuclear localization during larval feeding stages (1). The critical 20E titer upregulated PTEN expression to keep FoxO nucleus localization, which initiated PTP1B expression to dephosphorylate INSRβ (2) and repressed INSRβ expression (3). The decrease in INSR levels and phosphorylation weakened its inhibition on FoxO nuclear localization. Thereby 20E counteracted insulin pathway, stopped growth, initiated metamorphosis (larval cells apoptosis and adult tissues proliferation), and accumulated hemolymph glucose levels for imaginal discs growth in ILPs reach condition. 5F, fifth instar feeding larvae (about fifth instar 0 h–36 h); 5M, fifth instar molting larvae (about fifth instar 36 h–56 h); Adult, the whole body of adult; F, feeding; M, larval molting; MM, metamorphic molting; P, pupae; P 0–P 8, 0-day-old to 8-day-old pupae; sixth-6 h–sixth-120 h, time stages of sixth instar larvae.

INSR functions to promote insect larval growth and 20E production to reach a critical titer during the larval feeding stages. The critical 20E titer counteracts INSR function by upregulating PTEN expression, which maintains FoxO’s nuclear localization to promote PTP1B expression, resulting in INSR dephosphorylation. FoxO also repressed INSR expression. The critical titer of 20E stops larval growth, determines the body size, triggers metamorphosis, and causes accumulation of hemolymph glucose for imaginal disc growth (Fig. 10).

## Experimental procedures

### Insects

*H. armigera* larvae were raised in the laboratory at 25 to 27 °C under a photoperiod of 14 h light/10 h dark, and the larvae were reared on a previously described artificial diet (59).

### Western blot

Total protein was extracted from cells or larvae. Equal amount of each sample (50 μg) was subjected to 7.5% or 12.5% sodium dodecyl sulfate-polyacrylamide gel electrophoresis (SDS-PAGE). The proteins were transferred to nitrocellulose membranes (0.22 μm) by electrotransfer. The membranes were blocked with blocking buffer (5% nonfat milk in TBST (0.02% tween in TBS)) for 1 h at room temperature. The membranes were then incubated in blocking buffer with primary antibody at 4 °C overnight. The monoclonal antibody ACTB Rabbit mAb (ACTB), Mouse anti GFP-Tag mAb (Anti-GFP), and Mouse anti His-Tag mAb (Anti-His) were purchased from ABclonal (Cat. AC026, AE012 and AE003). Anti-insulin receptor β Rabbit polyclonal Abs (pAbs) (INSRβ) and anti-P-AKT1 (Ser473) Rabbit pAb were provided by the Wan Lei biological company (Cat. WL02857, WLP001); Phospho-IGF-I Receptor β (Tyr1135/1136)/Insulin Receptor β (Tyr1150/1151) (19H7) Rabbit mAb (anti-p-INSRβ) were provided by Cell Signaling Technology (Cat. 3024). The anti-p-INSRβ antibody was diluted with 5% BSA in 1:1000; other antibodies were diluted with 5% skim milk at 1:1000. After washing three times with TBST for 10 min each, the membranes were incubated with 1:7000 diluted secondary antibody, Goat Anti-Rabbit/-Mouse IgG-HRP conjugate secondary antibody (ZSGB-BIO). After washing three times with TBST and TBS, the membrane was immersed in High-sig ECL Western Blotting Substrate (Tanon Science & Technology). The chemiluminescent signal was detected using a 5200 Chemiluminescence Imaging System (Tanon Science & Technology). After exposure, the density of bands on the membrane was calculated using the ImageJ software. Acquired data were analyzed using GraphPad Prism 7 (GraphPad Software).

### Quantitative real-time reverse transcription PCR (qRT-PCR)

The total RNA extracted using the Trizol reagent (TransGen Biotech) was reverse transcribed into first-strand cDNA using M-MLV reverse transcriptase according to the manufacturer’s instructions (TIANGEN). qRT-PCR was carried out using a CFX96 real-time system (Bio-Rad) with 2 × SYBR qRT-PCR premixture (TransGen Biotech) and the primers listed in Table S1. *H. armigera* ACTB (GenBank accession no. EU52707) was used as the internal standard. The data represented the value of three biological replicates and were analyzed by the 2^−ΔΔCT^ method (ΔΔCT = ΔCT_treated sample_ − ΔCT_control_, ΔCT = CT_gene_ − CT_*Actb*_) (60). The relative mRNA levels of gene were used to express the expression levels of the gene by comparison with ACTB.

### λPPase treatment

We extracted the protein of the epidermis (consistent with our HaEpi cells) of the sixth instar 48 h and sixth instar 120 h larvae rapidly on ice (no phosphatase inhibitor was added during protein extraction to avoid inhibiting the activity of λPPase). Then, 40 μl of protein sample (2.5 mg/ml), 0.5 μl of λPPase, 5 μl of MnCl_2_, and 5 μl of buffer were incubated at 30 °C for 30 min, according to the manufacturer’s instructions (New England Biolabs). Next, the reaction products were examine using 7.5% SDS/PAGE gel followed by western blotting analysis. Phosphorylation of proteins was assessed according to their molecular weight changes.

### Enrichment of INSRβ protein

The larval tissues were homogenized in 500 μl of Tris-HCl buffer (40 mM, pH 7.5) on ice with 5 μl phenylmethylsulfonyl fluoride (PMSF, 17.4 mg/ml in isopropyl alcohol), respectively. The mixture was centrifuged for 15 min at 4 °C at 12,000 × *g*. The protein concentration of the supernatant was measured using the Bradford protein assay (61). Then, 2 μl of antibody against INSRβ and 50 μl of protein A beads were added to each 300 μg of tissue protein samples and incubated overnight at 4 °C. After centrifugation, the precipitates were suspended in 50 μl phosphate-buffered saline (PBS; 140 mM NaCl, 10 mM sodium phosphate, pH 7.4). Western blotting was then performed using the same level of INSRβ.

### Hormonal regulation of INSR

20E (Cayman chemical) and recombinant human insulin (P3376-400IU, Beyotime Biotech) were injected into the larvae, separately. 20E was first dissolved at a concentration of 10 mg/ml in DMSO (62), and the initial concentration of insulin was 4.5 mg/ml, which was diluted with PBS to a concentration of 100 ng/μl. Next, different concentrations of 20E and insulin were injected into sixth instar 6 h larvae with DMSO and PBS as controls. The concentration gradient of 20E was set to 100, 200, and 500 ng and that of insulin was set to 1, 2, and 5 μg. The same concentrations of 20E (500 ng) and insulin (5 μg) were injected at 1, 3, 6, 12, 24 h. The total mRNA or protein was extracted for qRT-PCR or western blot. HaEpi cells were treated with 5 μM 20E and 5 μg insulin for 1, 3, 6, 12, and 24 h to confirm the response of INSR to 20E and insulin induction, and equally diluted DMSO and PBS were used as solvent controls. HaEpi cells were treated with different concentrations of 20E and insulin for 6 h and then subjected to the same tests.

### Cell culture

The *H. armigera* epidermal cell line (HaEpi) was established from the *H. armigera* integument and has been well characterized previously (63). HaEpi cells were developed as a loosely attached monolayer and were cultured at 27 °C with Grace’s insect cell culture medium containing 10% fetal bovine serum (FBS) (Gibco).

### Double-stranded RNA (dsRNA) synthesis

RNA interference (RNAi) has been used widely in many species of moth (64). After the long dsRNA was broken down into smaller fragments *in vivo* (65), it specifically inhibits the expression of target genes in worms (66). After adding the T7 promoter to the RNAi primers (Table S1), the cDNA of the target gene was amplified by a single PCR to synthesize dsRNA. The dsRNA was synthesized in one reaction using a MEGAscript RNAi kit (Ambion) according to the manufacturer’s description. The product was purified using the phenol-chloroform method. After DNaseI digestion, the DNA template was removed. The dsRNA solution was brought to 200 μl using nuclease-free water, and the same volume of phenol-chloroform saturated water was added. After shaking the mixture for 15 s and centrifuging (9000 × *g*) at 4 °C, an equal volume of chloroform was added to the transferred upper aqueous phase and then centrifuged again. Next, 2.5 volumes of 100% ethanol and 1/10th volume of 3 M sodium acetate (pH 4.0) were added to the transferred supernatant and mixed overnight at −20 °C. We collected the dsRNA pellet by centrifugation the next day. The supernatant was discarded, and the pellet was washed with 75% ethanol, air-dried, and dissolved in 20 μl of nuclease-free water. The quality of the dsRNA was quantified using a microvolume spectrophotometer and detected using 1% agarose gel electrophoresis.

### RNA interference in HaEpi cells

In total, 4 μg of dsRNA was transfected into HaEpi cells for RNAi using the Quick Shuttle-enhanced transfection reagent (Biodragon Immunotechnologies) in 2 ml of Grace’s medium, according to the manufacturer’s instructions and our previous work (67). The control group received an equivalent amount of *dsGFP*. After 48 h of dsRNA treatment, 20E or an equivalent amount of DMSO was added to the cell culture medium at the final concentration required for the experiment, followed by an additional 6 h of incubation. Total RNA was then extracted from the cells for qRT-PCR analysis.

### Co-immunoprecipitation (Co-IP)

The reconstructed plasmids pIEx-4-PTEN-GFP-His or pIEx-4-PTP1B-GFP-His were transfected into the HaEpi cells. After 48 h, the cells were then treated with 2 μM 20E for 1 h. DMSO was used as the control. All experimental settings were treated with 5 μg insulin for 1 h as a control. Protein was subsequently extracted accordingly using radioimmunoprecipitation assay (RIPA) buffer with protease inhibitors, lysed on ice for 30 min, and then the supernatant was harvested *via* centrifugation at 12,000 × *g* for 30 min (4 °C). A small amount of the supernatant was used for western blotting analysis. Then, 30 μl of protein A agarose beads was washed three times with an appropriate amount of lysis buffer for 3 min. The supernatant was added to the protein A resin to eliminate nonspecific binding and harvested by centrifugation. The supernatant was then incubated with the protein A resin–antibody complex for 2 to 4 h with gentle shaking at 4 °C. The resin was subsequently harvested *via* centrifugation at 3000 × *g* for 3 min. The precipitate was washed with RIPA buffer three times. Finally, the resin was treated with SDS-PAGE loading buffer and boiled for 10 min. After centrifugation, the samples were subjected to SDS-PAGE, followed by western blotting analysis using mouse monoclonal antibody against GFP and anti-INSRβ Rabbit pAb to detect the target proteins.

### Overexpression of proteins in HaEpi cells

Plasmids encoding FoxO-GFP-His and GFP-His proteins were constructed in our laboratory (20). The ORFs of *Pten* (GenBank accession no. XP_021186139.1), *Ptpn1* (GenBank accession no. XP_021192174.1) were amplified by PCR using the corresponding primers (Table S1) and then inserted into an insect cell-specific overexpression vector (pIEx-4-GFP-His) to overexpress PTEN and PTP1B with C-terminal GFP and histidine tags, separately. *H. armigera* INSR contains a cleavage site (R-X-K/R-R, 785–788) (Fig. S8*B*), which would be cleaved by furin and separated as α and β subunits (12, 68). The actual molecular weight of INSRβ is higher than the theoretical molecular weight because it is highly glycosylated (13). Sites of potential asparagine-linked glycosylation in INSRβ were predicted by NetNGlyc 1.0 Server (http://www.cbs.dtu.dk/services/NetNGlyc/) (Fig. S8*B*). The ORF of *Insrβ* was amplified by PCR using the corresponding primers (Table S1) and then inserted into an insect cell-specific overexpression vector (pIEx-4-His) to overexpress INSRβ with C-terminal histidine tag. Sequencing was used to verify the correct PTEN, PTP1B, and INSRβ sequences. HaEpi cells were grown in six-well plates to 80% confluence, the medium was replaced with fresh medium, and 5 μg of plasmid was transfected into the HaEpi cells using the Quick Shuttle-enhanced transfection reagent. Subsequent experiments were carried out after 48 h.

### Immunocytochemistry

HaEpi cells were seeded at a density of 2 × 10^5^ in 500 μl of Grace’s medium supplemented with 10% FBS at 27 °C for 24 h. After FoxO-GFP-His was overexpressed for 24 h, 4 μg of dsRNA was transfected into the cells for 48 h. The cells were then incubated in PBS for 1 h and in insulin (5 μg/ml) for 6 h, followed by treatment with 5 μM 20E for another 6 h. HaEpi cells were washed three times with 500 μl Dulbecco’s phosphate-buffered saline (DPBS; 137 mM NaCl, 2.7 mM KCl, 1.5 mM KH_2_PO_4_ and 8 mM Na_2_HPO_4_, pH 7.4) and fixed with 4% paraformaldehyde diluted in PBS for 10 min in the dark at room temperature. The fixed cells were washed six times for 3 min each. Nuclei were stained with DAPI (1 μg/ml in PBS) (Sigma) in the dark at room temperature for 10 min. The negative control (GFP-His expression) was treated following the same method.

### Chromatin immunoprecipitation (ChIP)

The pIEx-4-FoxO-GFP-His plasmid was transfected into 80% density cells in six-well plates for 48 h. The cells were then treated with 20E for 6 h, and DMSO was used as the control. The ChIP Assay Kit (P2078, Beyotime Biotech) was used for the following assay. The cells were then cross-linked with 1% formaldehyde at 37 °C for 10 min. Glycine (0.125 M) was added to the mixture for 10 min to terminate the cross-linking. After washing the cells twice with PBS, SDS lysis buffer (1% SDS, 10 mM EDTA, 50 mM Tris-HCl, pH 8.1) was added to resuspend the cells. DNA was sheared into fragments of 200 to 1000 bp using a Bioruptor Pico (Diagenode). The samples were centrifuged at 4 °C for 15 min. Supernatants were added to 50 μl of protein A/G resin and incubated at 4 °C for 1 h to pretreat for nonspecific binding. After centrifugation, one supernatant was used as an input sample for qRT-PCR. Other supernatants were incubated with anti-GFP antibody or mouse control IgG as a negative control at 4 °C overnight. Then, 50 μl of protein A/G resin was added into the immunoprecipitated protein–DNA complex and incubated at 4 °C for 2 h. The complexes were washed once with low-salt wash buffer (0.1% SDS, 1.0% Triton X-100, 2 mM EDTA, 200 mM Tris-HCl, pH 8.0, 150 mM NaCl), followed by washing with a high-salt wash buffer (0.1% SDS, 1.0% Triton X-100, 2 mM EDTA, 20 mM Tris-HCl, pH 8.0, 500 mM NaCl), LiCl wash buffer (10 mM Tris-HCl, pH 8.1, 0.25 M LiCl, 1 mM EDTA, 1% NP-40, 1% deoxycholate), and twice with TE buffer (10 mM Tris-HCl, pH 8.1, 1 mM EDTA). The complexes were then washed with elution buffer (1% SDS, 0.1 M NaHCO_3_). The DNA–proteins structures were reverse cross-linked at 65 °C overnight, followed by RNase A and proteinase K treatments. The DNA was purified using phenol/chloroform extraction and analyzed by qRT-PCR with FoxOBE-F/FoxOBE-R primers (targeting FoxOBE) (Table S1).

### RNA interference (RNAi) of genes in larvae

The larvae that had grown to the required age (fifth instar 24 h or sixth instar 6 h) were placed on ice for 15 min until they did not move. The newly synthesized dsRNA was diluted to 300 ng/μl with PBS. In total, 5 μl of the diluted dsRNA was injected into the larval hemocoel from the side of the front abdomen with a sterile microsyringe. In total, 1.5 μg of dsRNA was injected into per larva. dsRNA was injected in 24 h intervals. Controls were treated with the same amount of *dsGFP*. Total RNA or protein was extracted to detect the effects of RNAi at 24 h after the last injection. Thirty larvae were injected for each treatment, and three independent replicates were performed.

### Detection of the 20E titer

The hemolymph was collected from at least three larvae at 66 h after the last injection of dsRNA. N-Phenylthiourea (10^−5^ μM, Aladdin) was added to each sample to prevent hemolymph blackening. The samples were centrifuged at 3000 × *g* for 5 min at 4 °C, and a 50 μl supernatant aliquot was freeze-dried for 3 h. Then, 80% methanol was added to extract the 20E, and the sample was evaporated until it dried completely at 4 °C. The pellet was dissolved in 100 μl enzyme immunoassay (EIA) buffer, and a 1 μl of the mixture was added into 1 ml EIA buffer. A 50 μl sample was used to detect 20E using a 20E Enzyme Immunoassay (20E-EIA) kit according to the instructions and operation manual (Bertin Pharma).

### Hemolymph glucose determination

More than three larvae, pupae, or adults at different developmental stages were collected, and then the second pair of feet was excised to collect about 30 μl of hemolymph per age. N-phenylthiourea was added to each sample to prevent hemolymph blackening. The samples were centrifuged at 3000 × *g* for 5 min at 4 °C, and 10 μl of the supernatant was used for hemolymph glucose determination according to the instructions of the glucose assay kit (Enzyme-linked Biotechnology Co, Ltd). The intensity of the color was determined at 505 nm by spectrophotometry (Infinite M200PRO NanoQuant, Tecan).

### Statistical analysis

All data were from at least three biological replicates. Student’s *t*-test was used to analyze two-group data sets. An asterisk represents a significant difference (*p* < 0.05), and two asterisks represent extremely significant differences (*p* < 0.01) in the figures. ANOVA was performed to evaluate the difference among three or more comparisons followed by post-hoc Tukey’s HSD test. In the ANOVA analysis, different letters represent significant differences (*p* < 0.05). The bars indicate the mean ± SD of three biological replicates. Figures were produced using GraphPad Prism 7.0. The western blotting results were analyzed using ImageJ software (National Institutes of Health, http://imagej.nih.gov/ij/download.html), and the data were derived from three independent replicates.

### The antibodies used in this study

The polyclonal antibody, anti-insulin receptor β Rabbit pAb (INSRβ), and anti-P-AKT1 (Ser473) Rabbit pAb were provided by the Wan Lei biological companies (Cat. WL02857, WLP001); the monoclonal antibody Phospho-IGF-I Receptor β (Tyr1135/1136)/Insulin Receptor β (Tyr1150/1151) (19H7) Rabbit mAb (p-INSRβ) was provided by Cell Signaling Technology (Cat. 3024). The monoclonal antibody ACTB Rabbit mAb (ACTB), Mouse anti GFP-Tag mAb (Anti-GFP) and Mouse anti His-Tag mAb (Anti-His) were purchased from ABclonal (Cat. AC026, AE012, and AE003).

## Data availability

All data are contained within the article.

## Conflict of interest

The authors declare that they have no conflicts of interest with the contents of this article.
