# Supplementary material for: The steroid hormone 20-hydroxyecdysone counteracts insulin signaling via insulin receptor dephosphorylation
Source: J Biol Chem. 2021 Jan 20;296:100318. doi: 10.1016/j.jbc.2021.100318 (PMC7949120; doi:10.1016/j.jbc.2021.100318)
Supplement: Supplemental Figures S1–S8 and Table S1 [file mmc1.pdf]

The steroid hormone 20-hydroxyecdysone counteracts insulin signaling via insulin receptor dephosphorylation

Yan-Li Li, You-Xiang Yao, Yu-Meng Zhao, Yu-Qin Di and Xiao-Fan Zhao*

Shandong Provincial Key Laboratory of Animal Cells and Developmental Biology, School of Life Sciences, Shandong University, Qingdao, 266237, China

*Corresponding author: Xiao-Fan Zhao
E-mail: xfzhao@sdu.edu.cn

Running title: 20E induces dephosphorylation of INSR

Supporting information:
Supplemental Figures and Legends: Figure S1-S8
Supplemental Table 1: Table S1

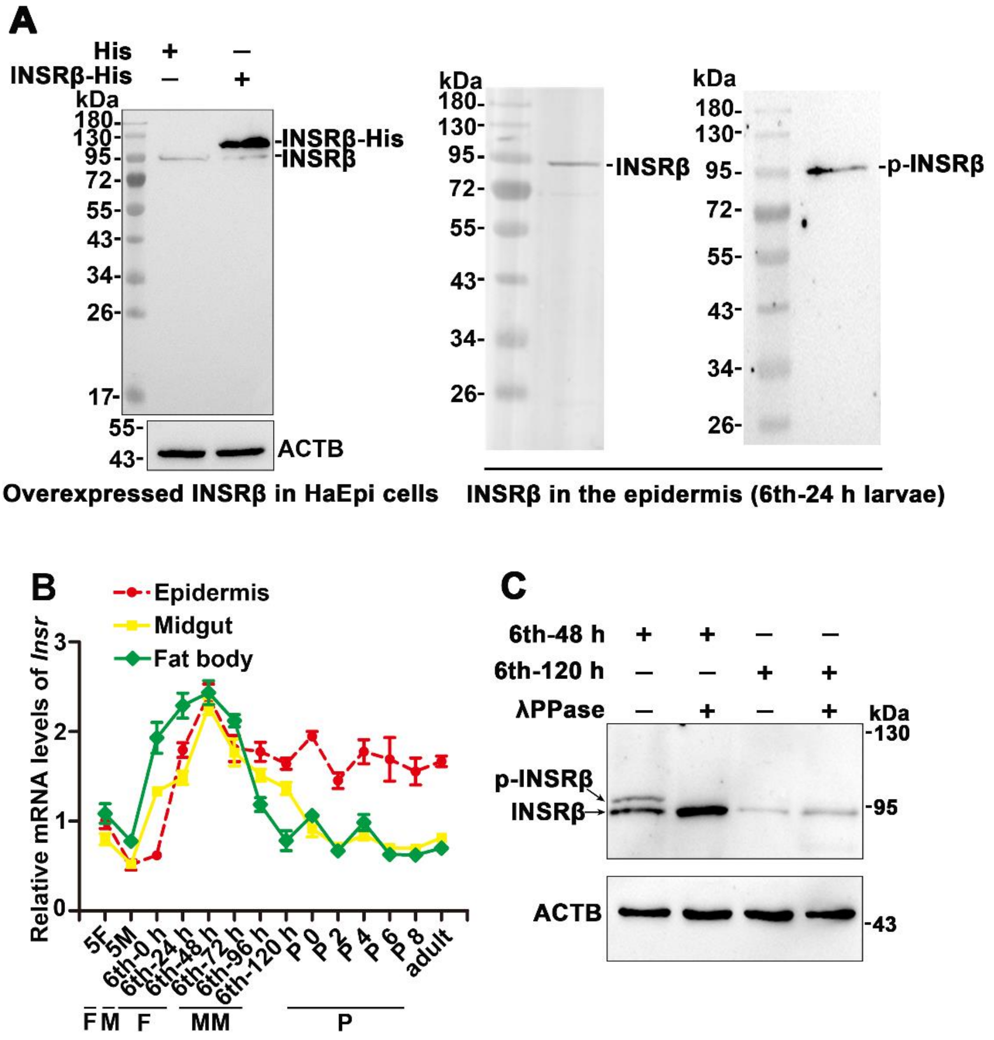

Figure S1. The control experiments for Figure 1. (A) The western blotting analysis of the antibody-specific detection. The INSRβ-His and His tag were overexpressed in the HaEpi cells for 48 h, respectively. INSRβ was detected by rabbit pAb anti-insulin receptor β (INSRβ), and p-INSRβ was detected by rabbit mAb anti-Phospho-insulin receptor β (the antibody diluted with 5% BSA in 1:1,000) as primary antibody. The secondary antibody was HRP labeled-Goat anti-rabbit. 12.5% SDS-PAGE gel. (B) qRT-PCR showing the expression profiles of *Insr* in various tissues during development, with *Actb* as the quantity and quality control. The relative mRNA levels were calculated by $2^{-\Delta\Delta CT}$, and the bars indicate the mean ± SD of three times repetition. The relative quantitative comparison was based on 5F epidermis. (C) λ-PPase decreased the intensity of the p-INSRβ band. 6th-48 h and 6th-120 h epidermis proteins were incubated with λ-PPase (0.5 μL λ-PPase in 50 μL buffer for 30 min at 30°C). SDS-PAGE gel in western blot was 7.5%. The polyclonal antibody against INSRβ was used for western blotting analysis. ACTB was used as the protein quantity control.

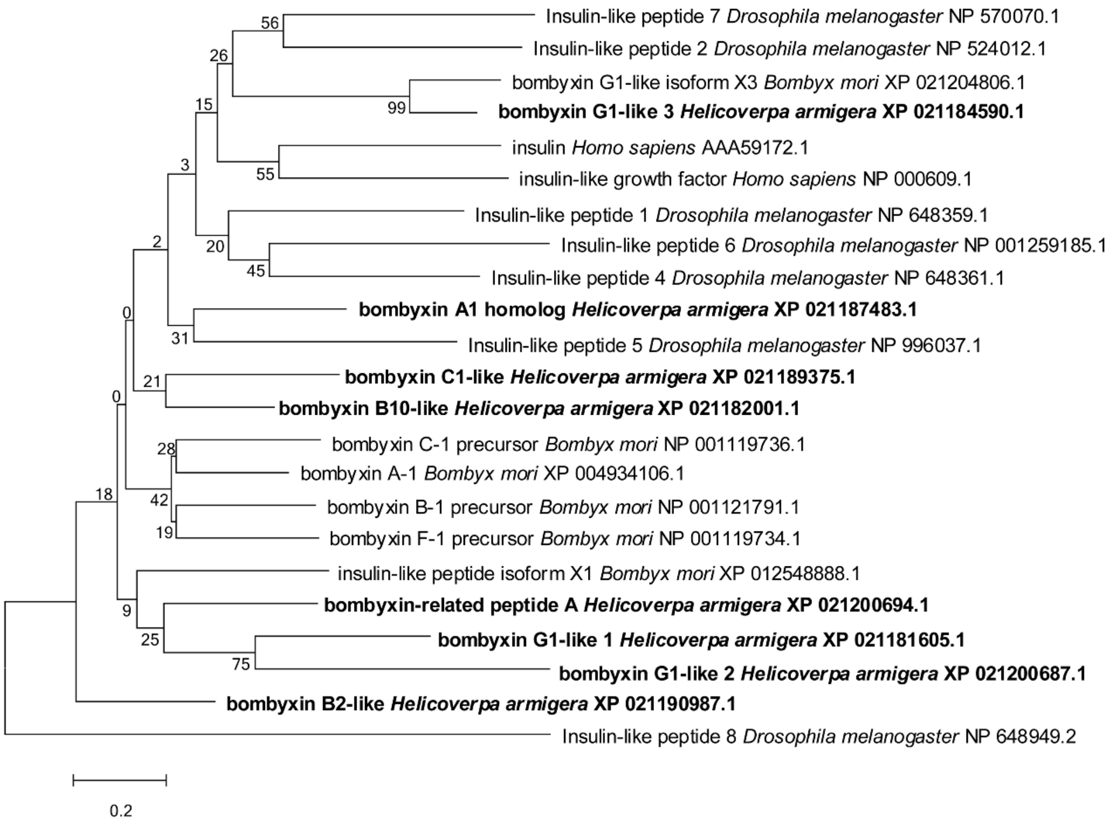

Figure S2. The phylogenetic analysis of ILPs of *H. armigera* and other organisms by MEGA 6.0 software. The numbers in the picture represent kinship levels. XP 021200687.1 and XP 021184590.1 were uncharacterized proteins in the genome and were identified as bombyxin G1-like 2 and bombyxin G1-like 3 here, respectively.

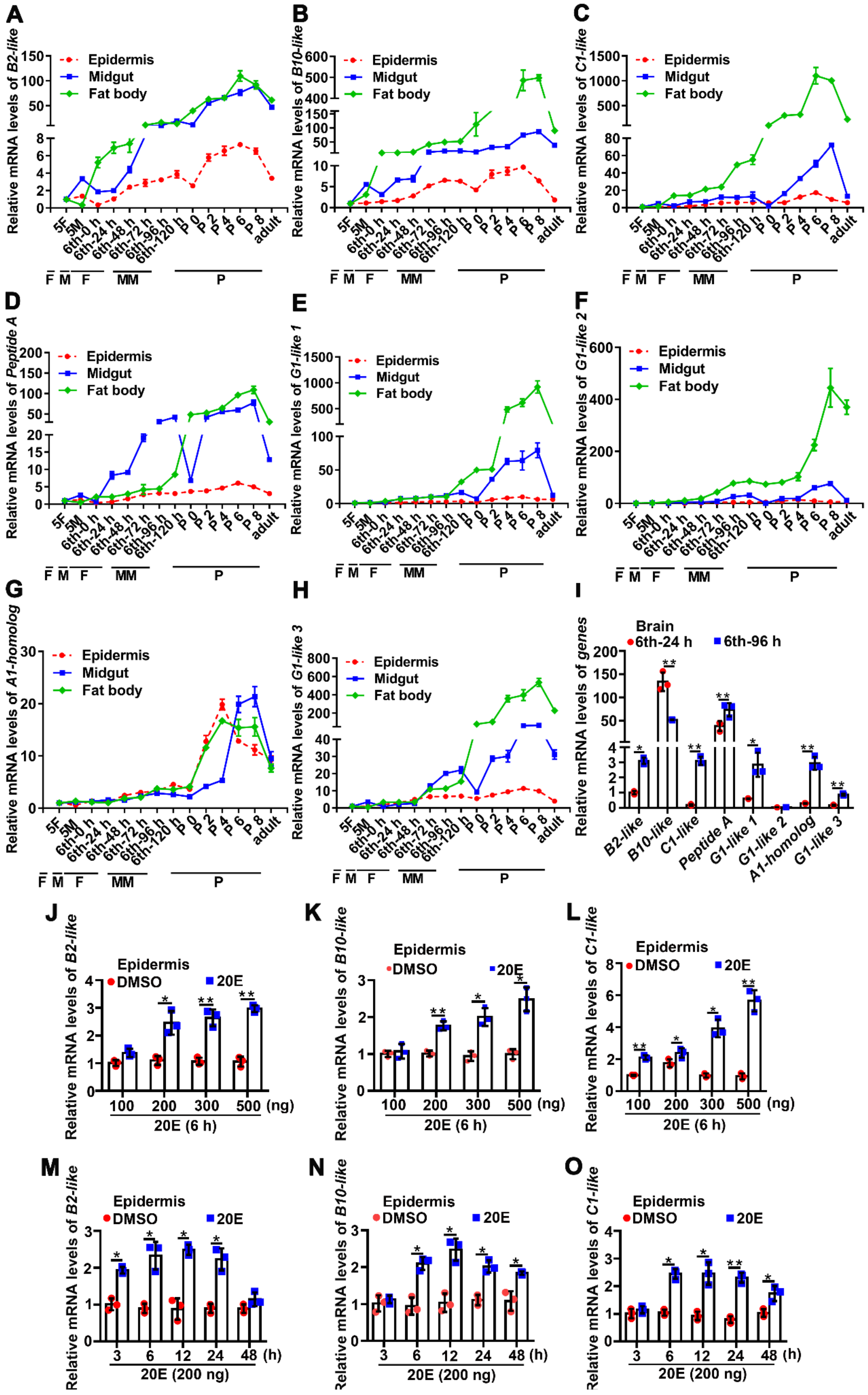
A
B
C
D
E
F
G
H
I
J
K
L
M
N
O
Epidermis
Midgut
Fat body
Relative mRNA levels of B2-like
Relative mRNA levels of B10-like
Relative mRNA levels of C1-like
Relative mRNA levels of Peptide A
Relative mRNA levels of G1-like 1
Relative mRNA levels of G1-like 2
Relative mRNA levels of A1-homolog
Relative mRNA levels of G1-like 3
Relative mRNA levels of genes
Brain
6th-24 h
6th-96 h
DMSO
20E
20E (6 h)
20E (200 ng)
(ng)
(h)
F M F MM P

Figure S3. qRT-PCR showing the expression profiles of *ILPs* and 20E induction. (A) to (H) qRT-PCR showing the expression profiles of eight ILPs in epidermis, midgut and fat body during larval development. The NCBI (https://www.ncbi.nlm.nih.gov/) reference sequence numbers of all ILPs are same as Fig. S2. The bars indicate the mean ± SD of three biological replicates (the total RNA was isolated from more than three insects in one repeat). (I) qRT-PCR showing the expression profiles of eight ILPs in brain at 6th-24 h and 6th-96 h. (J) to (O) Effect of 20E on the expression of *Ilps-B2-like*, *B10-like* and *C1-like* in epidermis by dose and time. Equal diluted DMSO was used as a control. All the relative mRNA levels were calculated by $2^{-\Delta\Delta CT}$, and the bars indicate the mean ± SD of three biological replicates. Significant differences were calculated using Student's *t*-test (**p* < 0.05, ***p* < 0.01) according to three biological replicates and three technical replicates in all qRT-PCRs in (I) to (O).

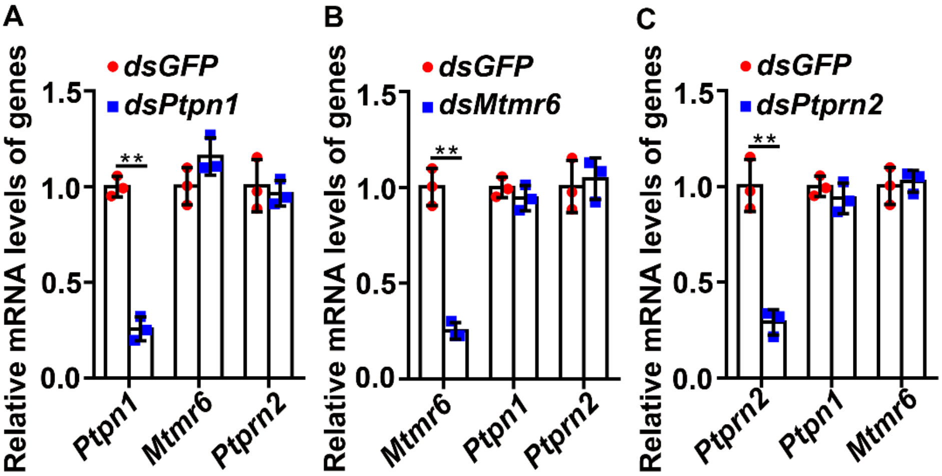

Figure S4. The efficacy and off-target detection of *PTPases* interference. Efficacy of *PTPases* knockdown in HaEpi cells by qRT-PCR analysis. (A) The relative mRNA levels of *Ptpn1*, *Mtmr6* and *Ptprn2* after knocking down of *Ptpn1*. (B) The relative mRNA levels of *Mtmr6*, *Ptpn1* and *Ptprn2* after knocking down of *Mtmr6*. (C) The relative mRNA levels of *Ptprn2*, *Ptpn1* and *Mtmr6* after knocking down of *Ptprn2*. All the relative mRNA levels were calculated by $2^{-\Delta\Delta CT}$, and the bars indicate the mean ± SD of three biological replicates. Significant differences were calculated using Student's *t*-test (***p* < 0.01).

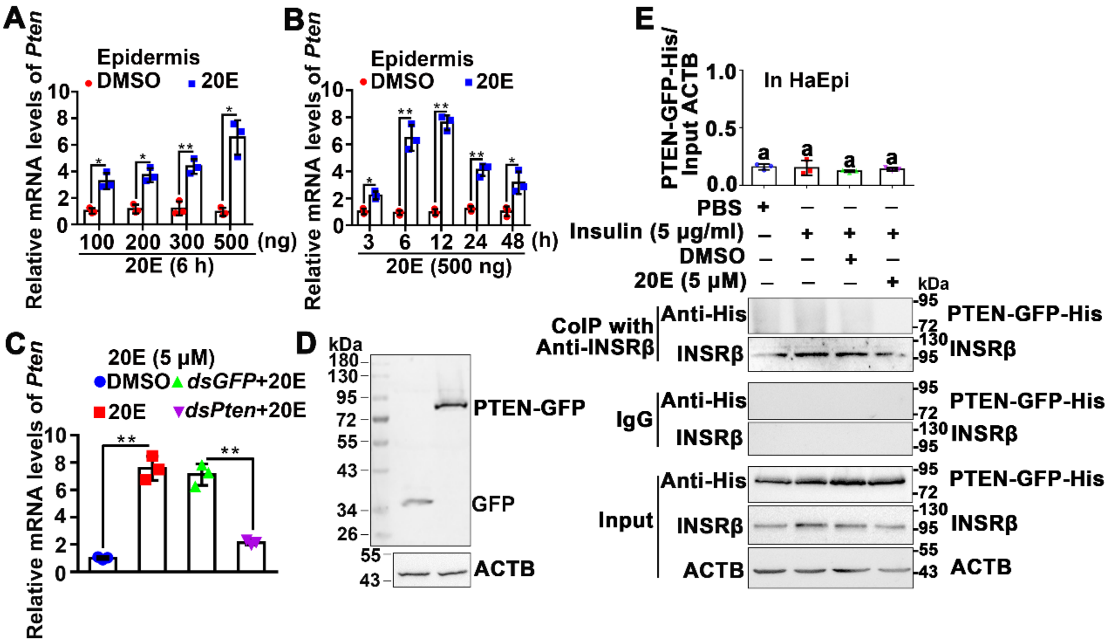

Figure S5. Co-IP experiments showing PTEN cannot interact with INSRβ. (A) and (B) qRT-PCR analysis of 20E regulation on *Pten* mRNA in larval epidermis by dose and time.

Equal volume of diluted DMSO was injected as control. (C) The efficacy of *Pten* knockdown by qRT-PCR analysis. DMSO was a solvent control for 20E. *dsGFP* was the control of *dsPten*. The relative mRNA level was calculated by $2^{-\Delta\Delta CT}$. The statistical analysis was performed using three times repetition by Student's *t*-test (**p* < 0.05; ***p* < 0.01) and the bars indicate the mean ± SD. (D) Efficacy of PTEN overexpression in HaEpi cells. 12.5% SDS-PAGE. (E) Co-IP of INSRβ and PTEN. The cells, after transfected with PTEN-GFP-His plasmid for 48 h, were incubated for 6 h with various reagents. The protein levels of INSRβ, PTEN-GFP-His and ACTB in HaEpi cells were detected via western blotting with 12.5% SDS-PAGE. INSR was immunoprecipitated with anti-INSRβ, and PTEN-GFP-His was detected using an anti-His mAb. Rabbit IgG was used as negative control of the antibody. Statistical analysis was conducted using ANOVA, different letters represented significant differences ($p < 0.05$). The bars indicate the mean ± SD of three replicates. ImageJ software was used to transform the image data.

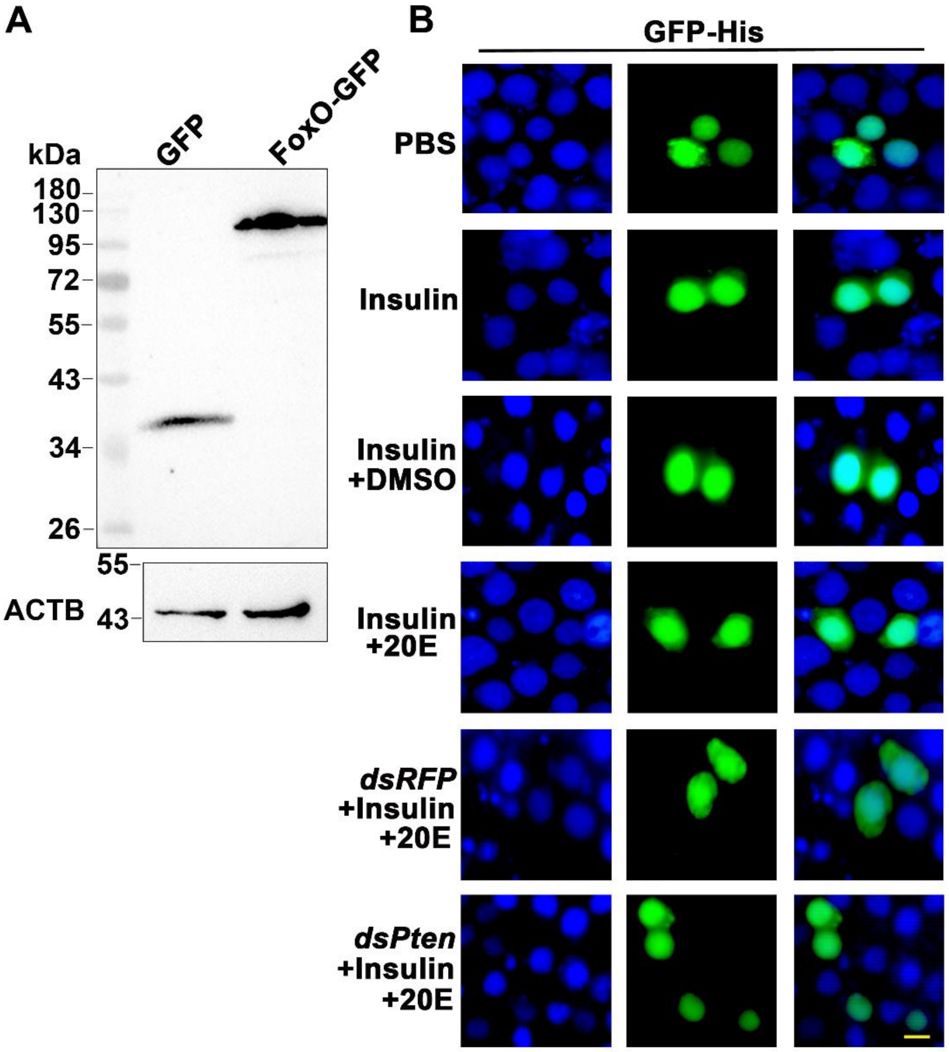

Figure S6. The subcellular localization of GFP-His tag was not affected by PTEN. (A) Overexpression of FoxO-GFP and the GFP tag in HaEpi cells for 48 h. Anti-GFP was used as primary antibody. The secondary antibody was HRP labeled-Goat anti-mouse. (B) The subcellular localization of GFP-His tag in the cells in various conditions (insulin 5 μg/mL, 20E 5 μM for 6 h, 4 μg of dsRNA for 48 h) without FBS. Green: green fluorescence from GFP. Blue: nucleus stained with DAPI. Scale bars: 20 μm.

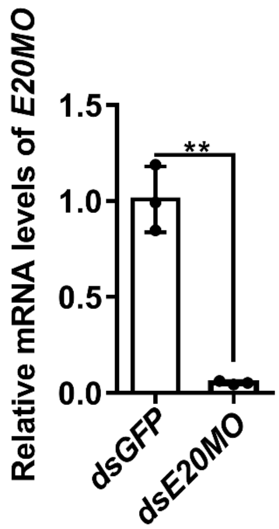

Figure S7. Efficiency analysis of *E20MO* knockdown after the last injection of *dsRNA* for 24 h using qRT-PCR. The relative mRNA levels were calculated by $2^{-\Delta\Delta CT}$. The statistical analysis was performed using three times repetition by Student's *t*-test (**p < 0.01). The bars indicate the mean ± SD.

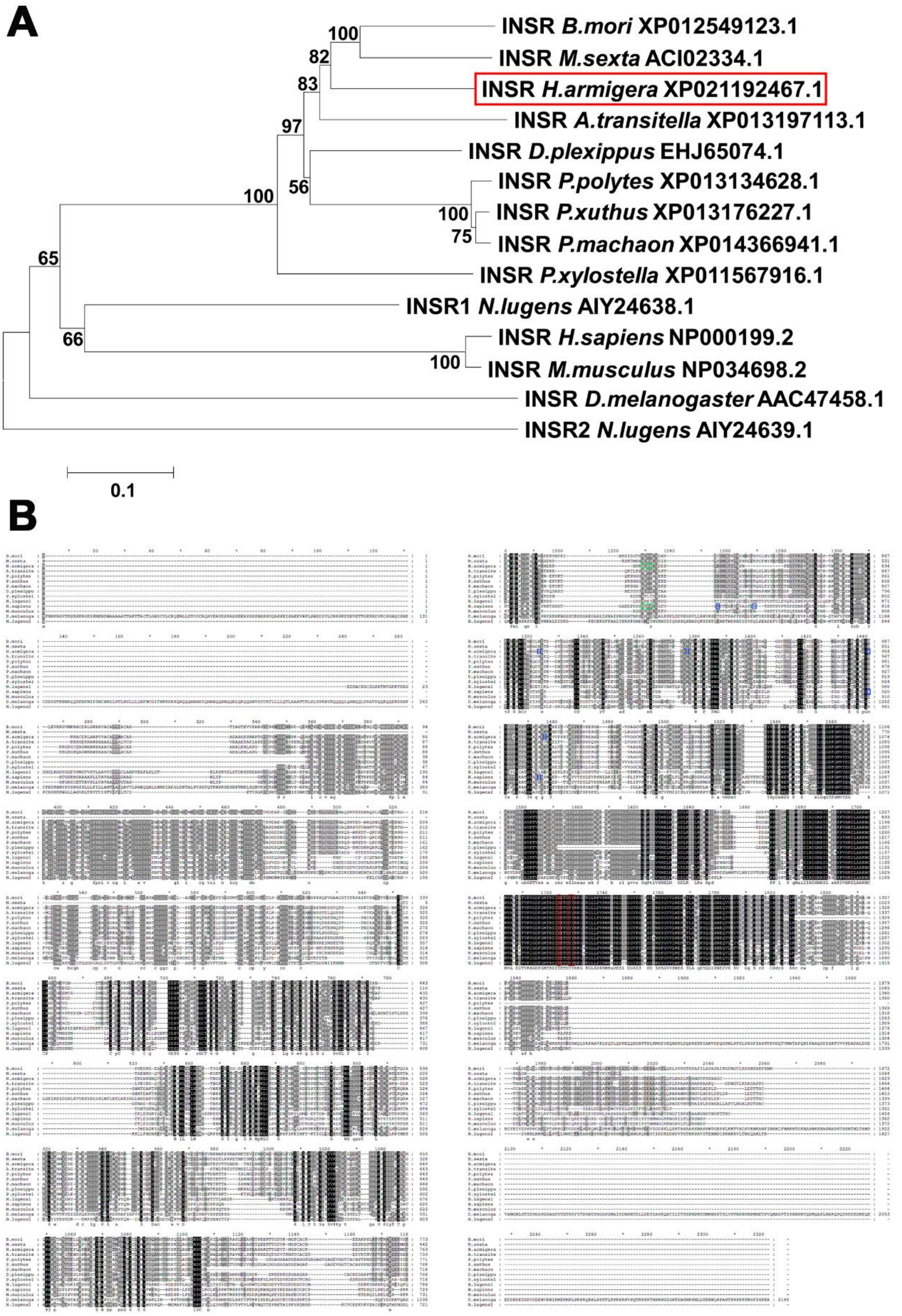

Figure S8. The bioinformatics analysis of INSR. (A) The phylogenetic analysis of INSR of *H. armigera* with the INSRs of other species by MEGA 6.0 software. The numbers in the picture represent kinship levels. *Nilaparvata lugens* has two kinds of INSR: INSR1 and INSR2. (B) The amino acid sequence of INSR of *H. armigera* was compared with the INSR amino acid sequences of the other species using GeneDoc software. The black area represents the conserved part of the INSR sequence. Three potential tyrosine phosphorylation sites (Tyr1146, Tyr1150, and Tyr1151) were marked with red boxes. The α/β furin cleavage sites were

marked with green boxes. Asparagines predicted to be N-glycosylated in INSRβ were highlighted in blue.

Table S1. Primers used in this study

Primer names	Sequence (5′-3′)
**Overexpression**	
PTEN-pIEx-F	tactcacaattggatgggtatttgcgtgagc
PTEN-pIEx-R	tactcaggcgcgccgatactcctgtggcgtggtg
FoxO-oex-F	tactcatactcagagctcatgtctatacggggcagc
FoxO-oex-R	tactcatactcaagatctggtggacccaggagggggc
PTP1B-oex-F	tactcagagctcatgagtcaaaacaacgtc
PTP1B-oex-R	tactcacaattgggcctaatttcctcagtctc
Insr-oex-F	tactcaggatccctcggtcgacgacagcctg
Insr-oex-R	tactcaggcgcgccgagcagccggcggcggaggg
**RNAi**	
GFP-RNAi-F	gcgtaatacgactcactataggtggtcccaattctcgtggaac
GFP-RNAi-R	gcgtaatacgactcactataggcttgaagttgaccttgatgcc
RFP-RNAi-F	gcgtaatacgactcactataggcttcgcctgggacatcct
RFP-RNAi-R	gcgtaatacgactcactataggggtgtagtcctcgttgtggg
Insr-RNAi-F	gcgtaatacgactcactataggctttcaacaccttccgcacta
Insr-RNAi-R	gcgtaatacgactcactataggcacccaggactaagaacattatcat
Pten-RNAi-F	gcgtaatacgactcactataggcggctgactccagaaatg
Pten-RNAi-R	gcgtaatacgactcactataggtatcatccaaggcaggta
FoxO-RNAi-F	gcgtaatacgactcactataggcaagacaacagactcacg
FoxO-RNAi-R	gcgtaatacgactcactataggttgtccgaagtccgtttg
Ptpn1-RNAi-F	gcgtaatacgactcactataggtggcaacctgaatctgtc
Ptpn1-RNAi-R	gcgtaatacgactcactataggttctttcttatcgtcctcc
Mtmr6-RNAi-F	gcgtaatacgactcactataggagcgttctcccgtcttcac
Mtmr6-RNAi-R	gcgtaatacgactcactataggtgttcttatccgccttcttac
Ptprn2-RNAi-F	gcgtaatacgactcactataggcctgaaccaagggtctacatt
Ptprn2-RNAi-R	gcgtaatacgactcactataggcggctcctatcaccaacactaac
E20MO-RNAi-F	gcgtaatacgactcactataggaccatcttcgtcgccacct
E20MO-RNAi-R	gcgtaatacgactcactatagggcagccttcttgtccctca
**qRT-PCR**	
Insr-F	tcttggtacaccgtgaacatc
Insr-R	actacgaagccgttggggttctgag
Pi3k-F	tggaggagttcacgatga
Pi3k-R	cccttctgtcccttattg
Akt-F	gcaacaaacagcgacaggc
Akt-R	ccgtcaatcgggtctaca
B10-like-F	ggttctcgctatcgtgg
B10-like-R	ggtttgtagcagcactcg
B2-like-F	tgattctcattgtcgctgtc
B2-like-R	gcttgccacgcatacc
C1-like-F	gctgctggtagtagtgtcg
C1-like-R	aggcagcactcgtcca

Peptide A-F	gttatggctccgactatg
Peptide A-R	aggtttgaggcagcac
G1-like 1-F	cgccaagttcgctatt
G1-like 1-R	cagacggtaaatcactaatc
G1-like 2-F	gtaggaggcgactggtt
G1-like 2-R	cagcagtttcgcatcg
A1-homolog-F	tgtgccctgatgacggag
A1-homolog-R	tgacgacgaacctcttgc
G1-like 3-F	acaaggcagttcagttcg
G1-like 3-R	gtcagcaataccgtttcc
Pten-F	tcttccacttctggttca
Pten-R	gtgtttatgctgcttatcc
β-actin-F	cctggtattgctgaccgtatgc
β-actin-R	ctgttggaaggtggagagggaa
FoxO-F	tcattacccaagccagcac
FoxO-R	tccatccagccgaagagt
Ptpn1-F	tcaacatccaggagacgctt
Ptpn1-R	atgacggcttggtagcagaa
Mtmr6-F	cagagaatgaatgtgcctaacg
Mtmr6-R	ctcgcttgggtatgtgtcg
Ptprn2-F	acaacccacccctaacacca
Ptprn2-R	cttctgggcaaggattcgtt
E20MO-F	ggaggtgcttctgtggtgt
E20MO-R	cgtatctgtcgggtctgct
**CHIP**	
FoxOBE-*Ptpn1*-F	cgaacggagcgacatatctaa
FoxOBE-*Ptpn1*-R	tccaccaatgggaggtttact
FoxOBE-*Insr*-F	ctaaaccaacgaggcattcaagt
FoxOBE-*Insr*-R	gtattaccgatagcaaatccaag
